# Defect Engineering Activates Pyrene‐Based Covalent Organic Frameworks for Efficient Photocatalytic Uranium Capture

**DOI:** 10.1002/advs.202518948

**Published:** 2025-12-23

**Authors:** Weikun Yao, Guanbing Zhou, Tao Liu, Yihui Yuan, Hui Wang, Ning Wang

**Affiliations:** ^1^ State Key Laboratory of Marine Resource Utilization in South China Sea Hainan University Haikou 570228 China

**Keywords:** covalent organic frameworks, defect engineering, hydrophilicity, photocatalysis, uranium capture

## Abstract

Efficient uranium capture from wastewater is essential for environmental remediation and sustainable nuclear energy, yet current adsorption and photocatalytic approaches remain limited by insufficient active sites, charge recombination, and hydrophobicity. Herein, a defect‐engineered pyrene‐based covalent organic framework (TP‐PYTO‐SA) is reported, synthesized from 2,4,6‐triformylphloroglucinol (TP) and 2,7‐diaminopyrene‐4,5,9,10‐tetraone (PYTO) with strategic salicylaldehyde (SA) incorporation. The introduced phenolic hydroxyl groups of SA improve hydrophilicity and offer additional uranyl binding sites, while rational defect density optimization (20%) generates localized electron traps. Concurrently, the electron‐donating effect of phenolic hydroxyl groups establishes a built‐in electric field, which significantly facilitates charge separation and enables a precisely tailored bandgap of 1.70 eV. Under standard AM 1.5G solar irradiation, TP‐PYTO‐SA_20_ enables outstanding photocatalytic uranium extraction without a sacrificial agent, achieving an impressive uranium adsorption capacity of 1151.65 mg g^−1^, 38.82% higher than pristine TP‐PYTO. It demonstrates excellent recyclability (> 90% efficiency retention after 10 cycles) and maintains 95.47% uranium removal in competing‐ion‐containing simulated wastewater. This work highlights defect engineering via tailored monomer substitution as a versatile paradigm for designing advanced covalent organic frameworks (COFs) for photocatalytic applications in radioactive pollution control and resource recovery.

## Graphical abstract

A defect‐engineered pyrene‐based covalent organic framework (TP‐PYTO‐SA) is developed via strategic salicylaldehyde incorporation. This approach simultaneously enhances hydrophilicity and introduces uranyl coordination sites, while optimized defect density significantly improves charge separation. Under illumination, TP‐PYTO‐SA_2_₀ achieves an exceptional uranium adsorption capacity of 1151.65 mg g^−1^ and 95.47% removal efficiency in simulated nuclear wastewater, demonstrating outstanding reusability and selectivity for sustainable uranium capture.

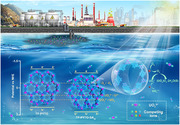

## 1 Introduction

Nuclear energy, as an efficient and low‐carbon alternative to fossil fuels, plays a vital role in the global energy transition.^[^ ^1^, ^2^ ^]^ Uranium, the primary fuel for nuclear power, is indispensable in this process.^[^ ^3^ ^]^ However, its mining and the reprocessing of spent nuclear fuel inevitably generate large amounts of uranium‐containing wastewater.^[^ ^4^, ^5^ ^]^ This not only threatens environmental safety but also represents a substantial loss of valuable resources, thereby creating dual challenges of remediation and recovery.^[^ ^6^, ^7^ ^]^ Adsorption has been widely regarded as a practical treatment strategy owing to its operational simplicity and cost‐effectiveness.^[^ ^8^ ^]^ Nevertheless, the adsorption capacity is inherently restricted by the finite number of coordination sites on adsorbents, limiting its efficiency for application.^[^ ^9^ ^]^ Recently, photocatalytic adsorption–reduction cascade technology has emerged as a promising alternative. Through light‐driven processes, soluble U(VI) ions captured on functional groups can be reduced to insoluble U(IV) precipitates, which subsequently detach and regenerate active sites, enabling continuous uranium capture and fundamentally overcoming the site‐limitation bottleneck. Despite this progress, achieving efficient charge separation, fast interfacial electron transfer, and high selectivity in complex aqueous systems remains challenging.^[^ ^10^ ^]^ Therefore, the development of advanced photocatalytic materials with high efficiency, selectivity, and sustainability is crucial for environmental remediation and the long‐term sustainability of the nuclear fuel cycle.^[^ ^11^ ^]^

COFs represent a pioneering class of porous crystalline polymers that enable precise assembly of organic building blocks into predesigned 2D or 3D architectures through robust covalent linkages.^[^ ^12^, ^13^, ^14^ ^]^ Owing to their exceptional characteristics, including low density,^[^ ^15^ ^]^ ultrahigh surface area,^[^ ^16^ ^]^ high crystallinity,^[^ ^17^ ^]^ extended π‐conjugation,^[^ ^18^ ^]^ narrow bandgaps,^[^ ^19^ ^]^ and tunable functionality, COFs have been widely applied in fields such as gas adsorption/separation, heterogeneous catalysis, chemical sensing, and energy storage.^[^ ^20^, ^21^ ^]^ In particular, pyrene‐based COFs stand out due to their extensive intrinsic π‐conjugation, which endows them with superior light‐harvesting capability, enhanced charge‐carrier mobility, and favorable band positions.^[^ ^22^, ^23^ ^]^ These attributes render them highly attractive photocatalytic platforms, demonstrating remarkable efficiency in photocatalytic reduction processes.^[^ ^24^ ^]^ However, their practical use in uranium extraction is hindered by two interrelated limitations. Intrinsic charge recombination rapidly annihilates photogenerated electron–hole pairs, severely reducing quantum yield and electron availability for U(VI) reduction.^[^ ^25^ ^]^ Meanwhile, the predominantly aromatic framework imparts pronounced hydrophobicity,^[^ ^26^ ^]^ restricting the diffusion of uranyl ions within the COF pores and hindering the contact between adsorption sites and U(VI) ions. These challenges underscore the need for rational structural engineering strategies to simultaneously promote charge separation and enhance hydrophilicity, unlocking the full potential of pyrene‐based COFs in photocatalytic uranium recovery.

Defect engineering has recently emerged as a powerful strategy for intentionally tailoring the physicochemical properties of crystalline materials.^[^ ^27^ ^]^ Representative approaches include generating high‐density atomic‐level defects in metal organic frameworks (MOFs) through ligand substitution to enhance electrocatalytic activity,^[^ ^28^ ^]^ and creating free amino groups in COFs via mixed‐monomer strategies for subsequent functionalization.^[^ ^29^ ^]^ Introducing controlled defects—such as vacancies,^[^ ^30^ ^]^ step sites,^[^ ^31^ ^]^ or heteroatom dopants—can profoundly alter electronic structure, surface chemistry, and mass transport characteristics.^[^ ^32^, ^33^ ^]^ This approach holds significant promise for enhancing the adsorption and photocatalytic performance of COFs. In the context of pyrene‐based COFs, strategic defect creation offers a compelling avenue to simultaneously address the twin challenges of insufficient photocatalytic activity and poor hydrophilicity. Defects can act as traps for charge carriers, significantly suppressing recombination and boosting the lifetime and concentration of photogenerated electrons available for U(VI) reduction.^[^ ^34^, ^35^ ^]^ Concurrently, defects, especially those terminating with polar functional groups or creating unsaturated coordination sites, can dramatically enhance the material's hydrophilicity.^[^ ^36^, ^37^ ^]^ This improved aqueous compatibility facilitates superior wetting, promotes intimate contact between the photocatalyst surface and U(VI) ions in solution, and enhances diffusion kinetics toward active sites.^[^ ^38^, ^39^ ^]^ However, excessive defect introduction may compromise the structural integrity of the COF framework, underscoring the need to carefully optimize defect density to achieve maximum uranium capture performance.^[^ ^40^, ^41^ ^]^

**Scheme 1:**
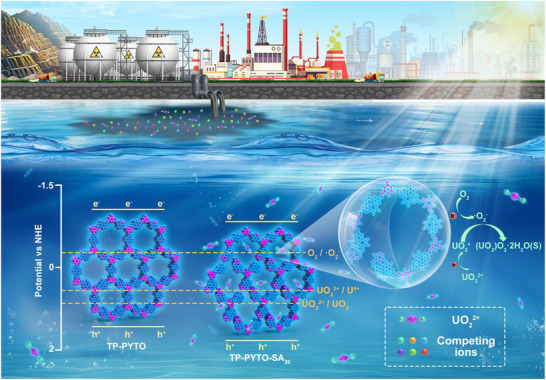
Schematic diagram of TP‐PYTO‐SA_20_ photocatalytic reduction mechanism of uranium.

In this study, we synthesized a defect engineering activates pyrene‐based COF (TP‐PYTO‐SA) using 2,7‐diaminopyrene‐4,5,9,10‐tetraone (PYTO), 2,4,6‐triformylphloroglucinol (TP), and salicylaldehyde (SA) as building units. During the polycondensation process, partial substitution of TP by SA, which contains only a single aldehyde group, introduced missing linkage sites and thereby generated structural defects in the framework.^[^ ^42^ ^]^ By varying the proportion of SA (10%, 20%, 30%), the defect density was modulated while maintaining the structural integrity of the COF (Figure S1, Supporting Information). Among these samples, TP‐PYTO‐SA_20_ with an optimized structure exhibited significantly enhanced photocatalytic activity while maintaining excellent crystallinity. Meanwhile, the inherent phenolic hydroxyl group of SA markedly improved the hydrophilicity of the framework, promoting strong interfacial interactions with aqueous uranium species.^[^ ^43^, ^44^ ^]^ Consequently, TP‐PYTO‐SA_20_ achieved an excellent adsorption capacity of 1151.65 mg g^−1^ in a 200 ppm uranium solution and a uranium removal efficiency of 95.47% in uranium‐containing wastewater. These results indicate that partial substitution of COF monomers with SA triggers a synergistic effect of defect‐activated photocatalysis and improved hydrophilicity, thereby enabling highly efficient uranium capture (**Scheme** **1**). This strategy proposed in this study provides a generalizable approach for the development of high‐performance materials for radioactive pollution remediation and shows broad potential in nuclear sustainability and environmental safety.

## 2 Results and Discussion

### 2.1 Structural and Characterizations

**Figure 1:**
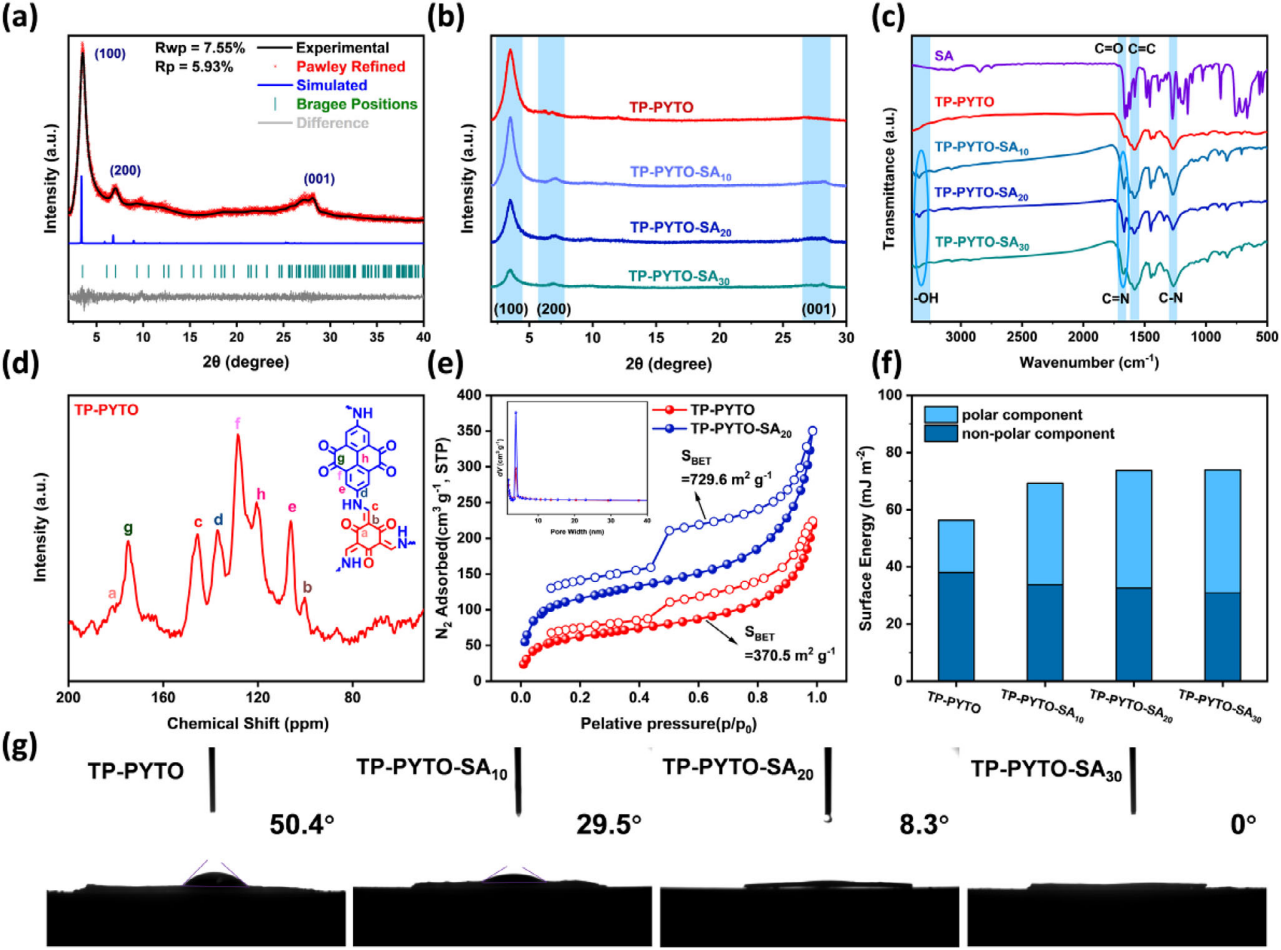
Structural characterization and physicochemical properties of TP‐PYTO and TP‐PYTO‐SAx. a) Experimental and Pawley‐refined PXRD of TP‐PYTO; b) PXRD patterns of TP‐PYTO, TP‐PYTO‐SA_10_, TP‐PYTO‐SA_20_, and TP‐PYTO‐SA_30_; c) FT‐IR spectra of TP‐PYTO, TP‐PYTO‐SA_10_, TP‐PYTO‐SA_20_, and TP‐PYTO‐SA_30_; d) Solid‐state 13C NMR spectra of TP‐PYTO; e) N_2_ adsorption‐desorption isotherms of TP‐PYTO and TP‐PYTO‐SA_20_; f) Apparent surface energy of different COFs; g) Water contact angle of TP‐PYTO, TP‐PYTO‐SA_10_, TP‐PYTO‐SA_20_, and TP‐PYTO‐SA_30_.

TP‐PYTO and TP‐PYTO‐SA_X_ with varying defect concentrations were synthesized via a solvothermal approach (Figure S2, Supporting Information),^[^ ^45^, ^46^ ^]^ as detailed in the Experimental Section of the Supporting Information, and their structural and physicochemical properties were systematically examined (**Figure** **1**). The crystal structure of TP‐PYTO was characterized by powder X‐ray diffraction (PXRD), combined with structural simulation and Pawley refinement (Figure 1a). The crystal structure of TP‐PYTO was characterized using powder X‐ray diffraction (PXRD), structural simulation, and Pawley refinement methods (Figure 1a). The characteristic diffraction peaks near 3.6°, 7.1°, and 27.7° correspond to the (100), (200), and (001) characteristic planes of TP‐PYTO, respectively, indicating excellent crystallinity. A structural model for the TP‐PYTO framework was established based on an eclipsed (AA) stacking conformation, and geometric and energy optimization were carried out using Materials Studio (Figure S3 and Tables S1, Supporting Information). The experimental PXRD spectra of TP‐PYTO are highly consistent with the simulated AA stacking model PXRD spectra, conforming to the P6/m space group of a hexagonal lattice (a = b = 30.075 Å, c = 3.524 Å, α = β = 90°, γ = 120°), with final Pawley refinement Rwp and Rp values of 7.55% and 5.93%, respectively. By comparing the PXRD spectra of TP‐PYTO and TP‐PYTO‐SA with different defect ratios, it can be found that as the defect ratio increases, the crystallinity of the COF material decreases to a certain extent. TP‐PYTO‐SA_10_ and TP‐PYTO‐SA_20_ still maintain strong diffraction peaks, but the diffraction peak intensity of TP‐PYTO‐SA_30_ on the (100) plane is only one‐fifth of the TP‐PYTO (Figure 1b).

Fourier‐transform infrared (FT‐IR) spectra conclusively verified the structural evolution across the COF series (Figures 1c; S4, Supporting Information), where the pristine TP‐PYTO spectrum exhibited complete disappearance of the precursor N–H stretching vibration (≈3300–3450 cm^−1^) alongside the emergence of definitive β‐ketoenamine signatures: a characteristic C–N stretching vibration (≈1280 cm^−1^), aromatic C═C stretching (≈1560 cm^−1^), and an intense β‐keto carbonyl (C═O) stretching (≈1620 cm^−1^). Crucially, the defect‐engineered TP‐PYTO‐SA manifested two diagnostically significant alterations: a phenolic O–H stretching vibration (≈3300 cm^−1^) originating from incorporated salicylaldehyde moieties, and critically, a persistent imine (C═N) stretching vibration (≈1640 cm^−1^). Notably, the intensity of the O–H band in TP‐PYTO‐SA_30_ is weaker than in its counterparts with lower SA loading. This apparent anomaly is reconciled by considering the severe structural degradation at high defect density. As confirmed by PXRD and N_2_ sorption, the framework of TP‐PYTO‐SA_30_ undergoes partial collapse and aggregation. This disordered, stacked morphology likely embeds a substantial fraction of phenolic hydroxyl groups, constraining their vibrational dynamics and thereby attenuating the FT‐IR response, despite the higher nominal concentration of SA. Additionally, solid‐state ^13^C nuclear magnetic resonance (^13^C NMR) spectroscopy further confirmed the successful incorporation of SA (Figures 1d; S5, Supporting Information). A distinct new resonance at 153 ppm emerged in the TP‐PYTO‐SA spectrum compared to pristine TP‐PYTO, unambiguously assigned to the ipso‐carbon of the phenolic hydroxyl group in SA.

On the basis of N_2_ adsorption‐desorption measurements at 77 K, the Brunauer Emmett Teller (BET) surface areas of TP‐PYTO, TP‐PYTO‐SA_10_, TP‐PYTO‐SA_20_, and TP‐PYTO‐SA_30_ were calculated to be 370.5, 488.3729.6, and 130.9 m^2^ g^−1^, respectively (Figures 1e; S6, Supporting Information). The BET surface area demonstrates a positive correlation with defect density; however, exceeding a critical defect threshold compromises structural integrity, resulting in pore collapse and a concomitant precipitous decline in BET surface area. In addition, the corresponding pore sizes are all 2.4 nm, which is highly consistent with the simulated pore size of the structure. The hydrophilicity of four covalent organic frameworks (COFs) was characterized by calculating their surface energy and comparing water contact angles (WCAs) (Figure 1f,g). Compared to TP‐PYTO, which exhibited a lower polar component in its surface energy, the introduction of defects led to a gradual increase in the polar component, while the dispersive component showed a slight decrease. This shift is attributed to the incorporation of phenolic hydroxyl groups from salicylaldehyde. Consequently, the water contact angle decreased significantly from 50.4° for TP‐PYTO to 0° for TP‐PYTO‐SA_30_, demonstrating the progressively enhanced hydrophilicity of the defective COFs. Sedimentation tests were performed to validate this conclusion (Figure S7, Supporting Information). When comparing TP‐PYTO and TP‐PYTO‐SA_20_, identical masses of each sample were dispersed in pure water. After 360 min of undisturbed standing, TP‐PYTO had completely settled at the bottom of the vial, whereas TP‐PYTO‐SA_20_ remained well‐dispersed in the aqueous phase.

**Figure 2:**
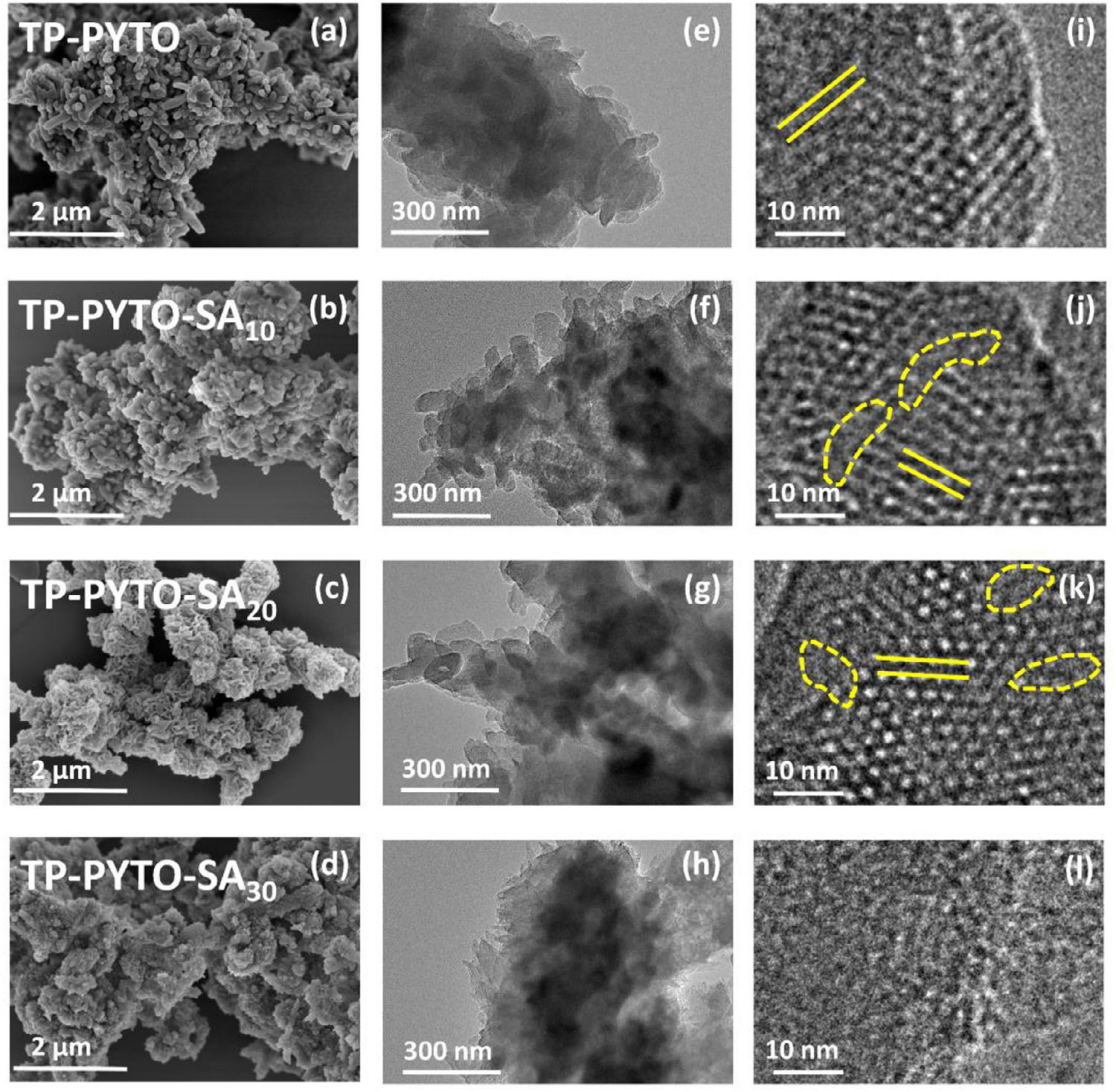
Morphological characterization of TP‐PYTO and TP‐PYTO‐SAx. a–d) SEM images of TP‐PYTO, TP‐PYTO‐SA_10_, TP‐PYTO‐SA_20_, and TP‐PYTO‐SA_30_; e–i) HR‐TEM images of TP‐PYTO; f–j) HR‐TEM images of TP‐PYTO‐SA_10_; g, k) HR‐TEM images of TP‐PYTO‐SA_20_; h–l) HR‐TEM images of TP‐PYTO‐SA_30_.

The morphological structures of the four COFs were analyzed using scanning electron microscopy (SEM) and transmission electron microscopy (TEM). SEM revealed that the pristine TP‐PYTO sample exhibits a well‐defined nanorod morphology with a smooth surface (**Figure** **2**a). In contrast, the TP‐PYTO‐SA samples with varying defect concentrations display a gradually increasing surface roughness on the nanorods (Figure 2b–d), providing indirect evidence for the successful incorporation of structural defects. HR‐TEM further confirmed the high crystallinity of TP‐PYTO, TP‐PYTO‐SA_10_, and TP‐PYTO‐SA_20_, as evidenced by well‐resolved lattice fringes (Figure 2e,i,f,j, and g,k). Notably, localized defect structures were clearly observed in TP‐PYTO‐SA_10_ and TP‐PYTO‐SA_20_ due to the incorporation of SA moieties. However, in the case of TP‐PYTO‐SA_30_, the significantly higher defect density substantially disrupted the long‐range atomic order, resulting in the absence of distinct lattice fringes (Figure 2h,l). This severe structural degradation correlates with the collapse of the porous framework, as supported by the markedly reduced BET surface area (130.9 m^2^ g^−1^) of TP‐PYTO‐SA_30_.

### 2.2 Photoelectric Activity

**Figure 3:**
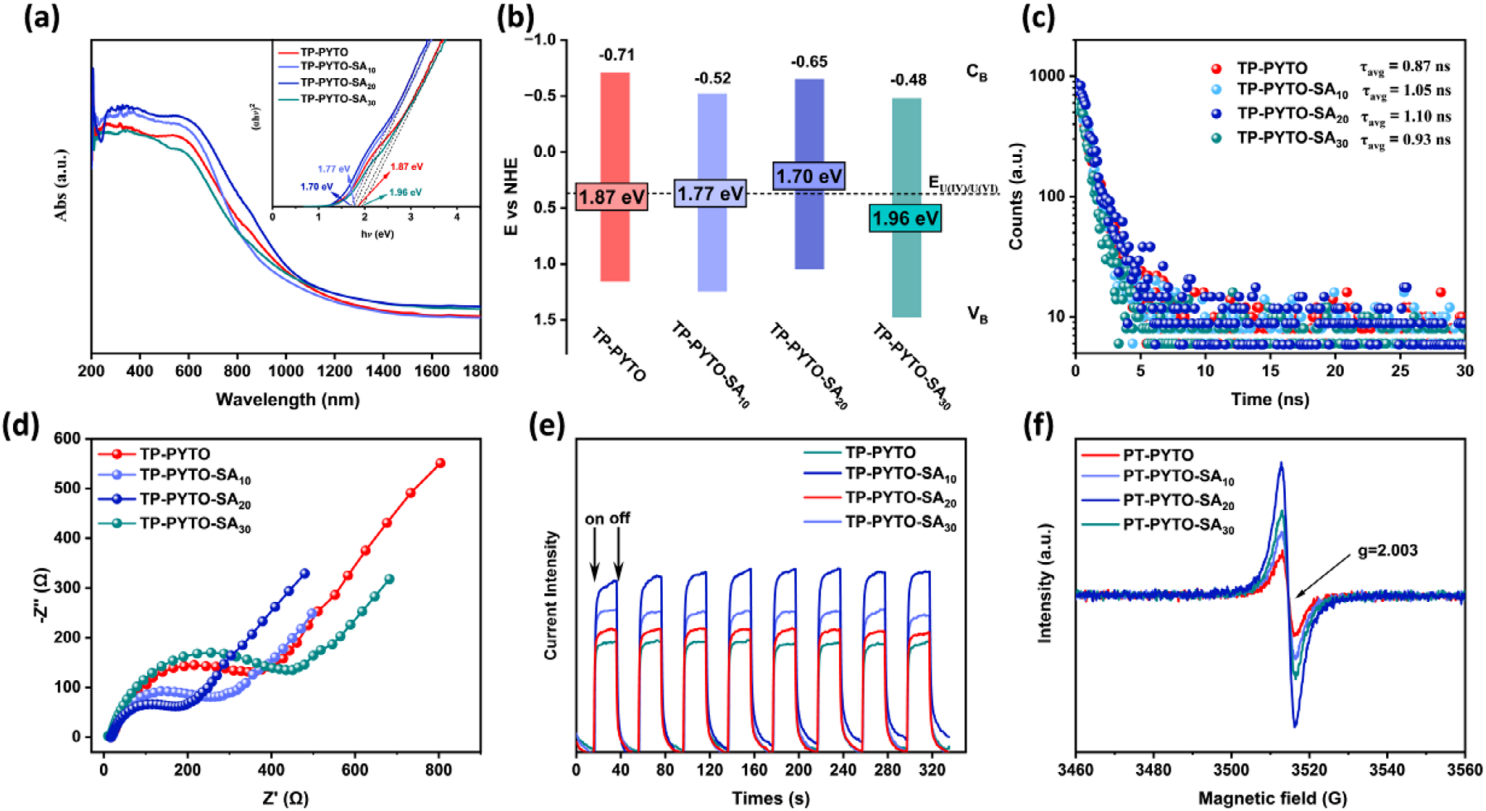
Photoelectrical characterization of TP‐PYTO and TP‐PYTO‐SAx. a) UV–vis DRS spectra; b) Band structure diagram; c) Time‐resolved PL spectra; d) Nyquist plots from EIS; e) Photocurrent response under chopped light illumination; f) EPR spectra.

Ultraviolet‐visible diffuse reflectance spectroscopy (UV–vis DRS) was employed to determine the optical band gaps (Eopt) of pristine and defect‐engineered COFs, evaluating their visible‐light harvesting capabilities.^[^ ^47^ ^]^ The UV–vis DRS profiles of all four COFs exhibited intrinsic absorption features within the visible spectrum (**Figure** **3**a). Compared to the pristine framework, TP‐PYTO‐SA_10_ and TP‐PYTO‐SA_20_ demonstrated significantly enhanced absorption edges across both UV and visible regions, while TP‐PYTO‐SA_30_ exhibited attenuated absorption onset. Optical band gaps derived from Tauc plots of Kubelka‐Munk (K‐M) transformed reflectance data were calculated as 1.87 eV (TP‐PYTO), 1.77 eV (TP‐PYTO‐SA_10_), 1.70 eV (TP‐PYTO‐SA_20_), and 1.96 eV (TP‐PYTO‐SA_30_) (Figure 3a, inset).^[^ ^48^ ^]^ Mott‐Schottky plots were measured at frequencies of 500, 1000, and 1500 Hz to estimate the conduction band (CB) energy levels (Figure S8, Supporting Information). Band structure comparisons demonstrate defect‐mediated band gap reduction in TP‐PYTO‐SA_10_ and TP‐PYTO‐SA_20_, confirming tailored electronic configurations (Figure 3b).^[^ ^49^, ^50^ ^]^ This phenomenon originates from the shallow trap states induced by the optimal SA concentration. These states enable sub‐bandgap excitation and, combined with the electron‐donating effect of ortho‐hydroxyl groups, facilitate the formation of a built‐in electric field (BIEF).^[^ ^35^ ^]^ The BIEF effectively drives the separation of photogenerated electron‐hole pairs and suppresses their recombination.^[^ ^51^ ^]^ Furthermore, the field‐induced force significantly enhances the free charge carrier flux, thereby substantially improving the overall photocatalytic efficiency.^[^ ^52^ ^]^ The synergistic interplay between this tailored BIEF and the optimized defect traps markedly enhances the separation and migration efficiency of photogenerated charges.

The attenuated emission intensity at 425 nm in the photoluminescence (PL) spectra of the three TP‐PYTO‐SA COFs indicates that defect incorporation suppresses the recombination of photogenerated carriers and enhances charge utilization efficiency, which isa crucial determinant for improving photocatalytic performance (Figure S9, Supporting Information). Time‐resolved fluorescence measurements yielded lifetimes of 0.87, 1.05, 1.10, and 0.93 ns for TP‐PYTO, TP‐PYTO‐SA_10_, TP‐PYTO‐SA_20_, and TP‐PYTO‐SA_30_, respectively (Figure 3c). In TP‐PYTO‐SA_20_, the presence of shallow defect traps effectively captures photogenerated charges and delays their recombination, while the BIEF further promotes the separation of these released charges, leading to a notably prolonged PL lifetime. To further elucidate their photoelectrochemical properties, electrochemical impedance spectroscopy (EIS) and transient photocurrent (I−t) were conducted. EIS analysis revealed that TP‐PYTO‐SA_20_ exhibited the smallest semicircular arc in Nyquist plots (Figure 3d), indicating the lowest charge transfer resistance (Rct) and most efficient charge transfer kinetics within the series.^[^ ^53^ ^]^ Equivalent circuit fitting of EIS spectra yielded Rct values of 345 Ω (TP‐PYTO), 253 Ω (TP‐PYTO‐SA_10_), 181 Ω (TP‐PYTO‐SA_20_), and 471 Ω (TP‐PYTO‐SA_30_) (Figure S10, Supporting Information). These results validate that strategically engineered structural defects at optimal concentrations facilitate enhanced charge transfer across the COF framework. Notably, TP‐PYTO‐SA_20_ demonstrated the highest and most stable photocurrent density across eight on‐off illumination cycles compared to the other COFs (Figure 3e). These collective results confirm that introducing SA defects at an optimal concentration effectively promotes the separation of photogenerated charges and suppresses electron‐hole recombination, thereby enhancing the photoelectrochemical performance.

Furthermore, upon light exposure, all COFs exhibited distinct electron paramagnetic resonance (EPR) signals (Figure 3f), confirming the generation of photochemically generated electron‐hole pairs within the frameworks, arising from photoexcited electrons in the conduction band under illumination compared to dark conditions (Figure S11, Supporting Information). The significantly enhanced paramagnetic resonance signal detected in TP‐PYTO‐SA_20_ provides direct evidence for a high concentration of unrecombined charge carriers under irradiation. These results collectively demonstrate that the 20% SA defect concentration optimally enhances charge carrier separation efficiency, thereby maximizing the population of free carriers available for photochemical processes. Notably, the decreased PL lifetime and EPR signal for TP‐PYTO‐SA_30_ indicate that excessive defect density becomes detrimental. Beyond the optimal level, defects evolve from beneficial shallow traps into deep traps that act as non‐radiative recombination centres, accelerating charge carrier annihilation and reducing the population of long‐lived photogenerated charges.^[^ ^54^ ^]^ This is consistent with the compromised crystallinity, collapsed porosity, and deteriorated photoelectrochemical performance of TP‐PYTO‐SA_30_, underscoring the necessity of defect density optimization.

### 2.3 Uranium Extraction Performance

**Figure 4:**
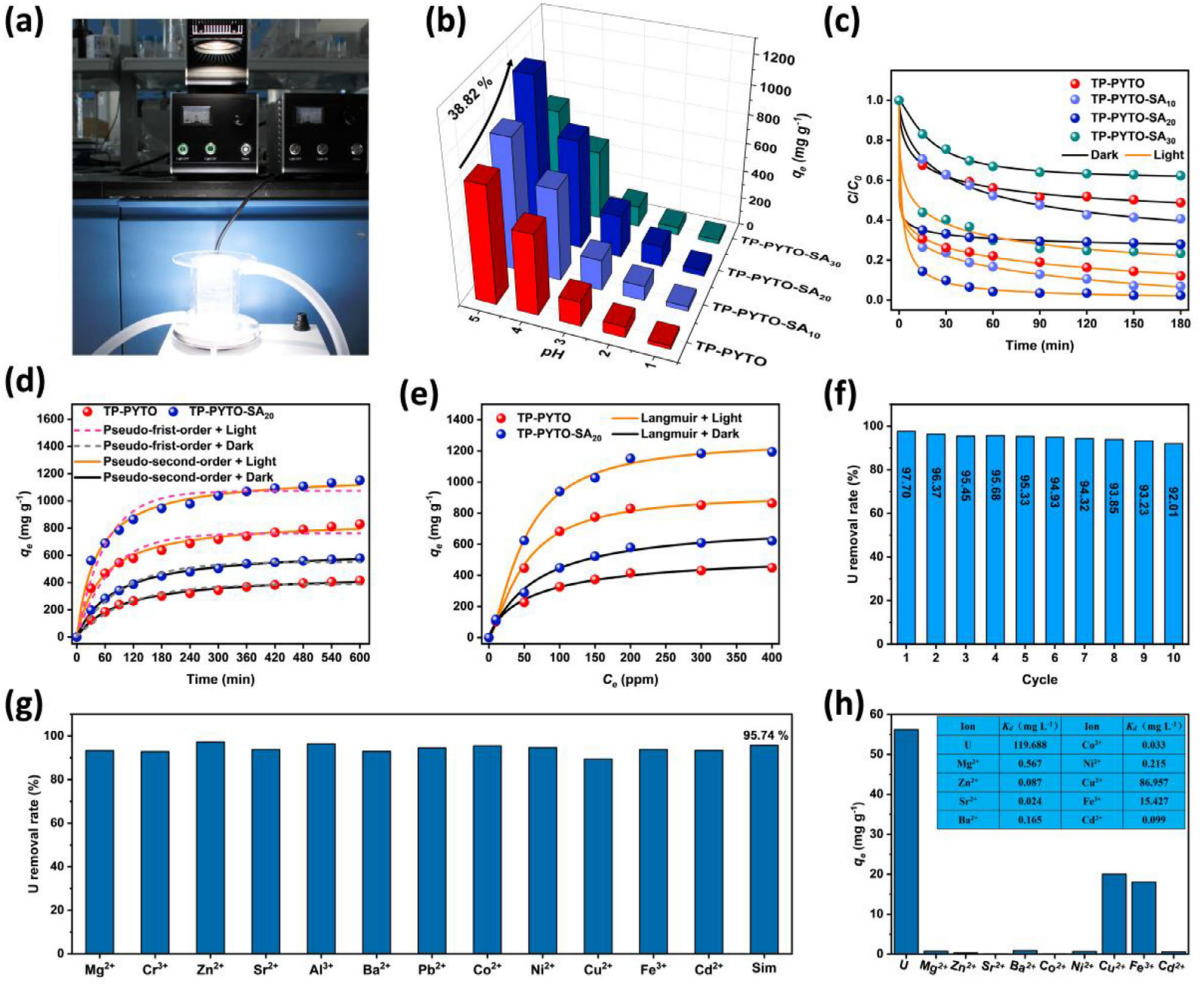
Adsorption properties of TP‐PYTO and TP‐PYTO‐SA_x_. a) Thermostated photocatalytic uranium adsorption system equipped with a xenon‐lamp solar simulator; b) Uranium adsorption capacity under simulated solar irradiation at pH 1.0–5.0; c) Uranium removal efficiency under light and dark conditions; d) Adsorption kinetics of TP‐PYTO and TP‐PYTO‐SA_20_ under light and dark conditions; e) Adsorption isotherms of TP‐PYTO and TP‐PYTO‐SA_20_ under light and dark conditions; f) Recyclability of TP‐PYTO‐SA_20_ for uranium photoreduction; g) Uranium removal efficiency of TP‐PYTO‐SA_20_ in the presence of competing ions and uranium‐containing wastewater; h) Adsorption capacities of coexisting ions in simulated uranium‐containing wastewater.

To evaluate the impact of defect‐engineered COFs on uranium extraction capability, photocatalytic adsorption experiments were performed under standard AM 1.5G solar irradiation (Xenon‐Lamp, 1 kW m^−2^) without sacrificial agents, with temperature maintained at 25 °C via the Thermostated Photocatalytic Uranium Adsorption System (**Figure** **4**a).^[^ ^55^, ^56^, ^57^ ^]^ Given the propensity of UO_2_^2^⁺ toward hydrolysis‐induced precipitation at pH > 5.0 and its predominant existence as hydroxyl‐coordinated U(VI) species with reduced average positive charge and diminished binding affinity (Figure S12, Supporting Information), pH‐dependent adsorption performance was systematically examined within the acidic regime (pH 1–5) (Figure 4b).^[^ ^58^ ^]^ All COFs exhibited progressively enhanced uranium uptake capacities with increasing pH, achieving maximum adsorption at pH 5. Remarkably, TP‐PYTO‐SA_20_ demonstrated the highest capacity (1151.65 mg g^−1^), representing a 38.82% enhancement over pristine TP‐PYTO (829.57 mg g^−1^). This electrostatic behavior, corroborated by zeta potential measurements (Figure S13, Supporting Information), revealed decreasing surface charge with increasing pH for both TP‐PYTO and TP‐PYTO‐SA_20_, reaching −4.13 and −5.29 mV at pH 5, respectively. These minimal negative values at the optimal pH indicate maximized electrostatic attraction between the COFs and UO_2_^2^⁺ species, facilitating access to specific binding sites. This compellingly demonstrates that optimal defect incorporation (20% SA) significantly enhances COF adsorption performance. Conversely, excessive defect loading in TP‐PYTO‐SA_30_ diminished its capacity below that of the unmodified framework.

Uranium removal kinetics were quantified under both dark and illuminated conditions (Figure 4c). Comparing the uranium removal efficiencies of the four COFs for uranium in solution, it can be clearly observed that, TP‐PYTO‐SA_20_ > TP‐PYTO‐SA_10_ > TP‐PYTO > TP‐PYTO‐SA_30_. In the dark, TP‐PYTO and TP‐PYTO‐SA_20_ achieved removal efficiencies of 51.2% and 70.3%, respectively. Under illumination, efficiencies increased substantially to 87. 9% for TP‐PYTO and 97.7% for TP‐PYTO‐SA_20_ (Tables S2, Supporting Information). TP‐PYTO‐SA_20_ exhibits superior removal performance under both dark and light conditions. Adsorption kinetics and isotherms for TP‐PYTO and TP‐PYTO‐SA_20_ were systematically studied (Figure 4d,e). To gain deeper mechanistic insights, the kinetic data were fitted with pseudo‐first‐order (PFO), pseudo‐second‐order (PSO), and intraparticle diffusion (IPD) models (Figure S14 and Tables S3–S5, Supporting Information). The PSO model yielded the highest correlation coefficients (R^2^ > 0.99) for both materials under light and dark conditions, and the calculated PSO rate constant (*k_2_*) for TP‐PYTO‐SA_20_ under light was 1.96 × 10^−5^ g mg^−1^ min^−1^, indicating that the adsorption process is predominantly governed by chemisorption.^[^ ^59^ ^]^ Furthermore, the IPO plot showed three distinct linear regions, revealing a multi‐stage process: the first sharp stage represents external surface adsorption, the second gradual stage is attributed to intra‐particle diffusion within the mesopores, and the third plateau stage corresponds to the final equilibrium. The second linear region did not pass through the origin (C ≠ 0), demonstrating that intra‐particle diffusion was not the only rate‐limiting step, which aligns with the chemisorption‐dominated mechanism. Equilibrium adsorption capacities increased with initial UO_2_^2^⁺ concentration, reaching 863.56 and 1194.49 mg g^−1^ for TP‐PYTO and TP‐PYTO‐SA_20_, respectively. The data conformed to the Langmuir isotherm model, confirming monolayer adsorption (Tables S6, Supporting Information).^[^ ^60^, ^61^ ^]^

To further clarify the relative contributions of adsorption and photocatalysis, we compared the maximum uranium uptake of TP‐PYTO and TP‐PYTO‐SA_20_ under both light and dark conditions (Figure 4d,e). Under dark conditions, TP‐PYTO‐SA_20_ consistently exhibits higher U(VI) uptake than pristine TP‐PYTO across the entire concentration range (∆q_e‐max_ = 172.43 mg g^−1^), confirming that defect engineering increases the density and accessibility of coordination sites and thus improves intrinsic adsorption. Under illumination, the performance gap between TP‐PYTO‐SA_20_ and TP‐PYTO becomes noticeably larger than that observed in the dark (∆q_e‐max_ = 330.93 mg g^−1^). This light‐induced enhancement consists of two parts: (i) the intrinsic adsorption gain already present in the dark, and (ii) an additional photo‐driven enhancement associated with accelerated U(VI) reduction. The larger improvement observed under illumination indicates that defect engineering strengthens not only adsorption but also the photo‐responsive reduction pathway. This conclusion is further supported by the photoelectrical characterization of TP‐PYTO and TP‐PYTO‐SA_x_ (Figure 3), which shows significantly improved charge separation and transfer efficiency in the defect‐engineered material. Therefore, the enhanced uranium removal performance of TP‐PYTO‐SA_20_ under both dark and illuminated conditions arises from a synergistic combination of effects: the incorporation of phenolic hydroxyl groups increases the number of effective UO_2_^2^⁺ coordination sites and enhances hydrophilicity to facilitate interfacial mass transfer, while the improved photoelectrochemical properties promote efficient charge‐mediated reduction processes under illumination. Together, these cooperative factors account for the superior performance of TP‐PYTO‐SA_20_.

Reusability was assessed through ten consecutive adsorption‐desorption cycles (Figure 4f). After each cycle, the material was regenerated by filtration, washing with 0.1 m HNO_3_ and ultrapure water, and vacuum drying before reuse. TP‐PYTO‐SA_20_ maintained exceptional performance, retaining over 90% uranium removal efficiency (92.01%) after the tenth cycle. The structural integrity of the recycled TP‐PYTO‐SA_20_ was further verified by PXRD and TEM (Figures S15 and S16, Supporting Information). The results indicate that the crystalline framework and surface morphology remained well preserved after reuse. The uranium selectivity of TP‐PYTO‐SA_20_ was evaluated at two levels to assess both its intrinsic affinity and its performance in a matrix simulating uranium‐containing wastewater. First, in a fundamental selectivity test with competing ions at a 3‐fold excess concentration, the material consistently achieved > 90% UO_2_^2^⁺ removal efficiency, demonstrating its high intrinsic affinity. More importantly, to assess its potential for practical wastewater remediation, TP‐PYTO‐SA_20_ was tested in a specially formulated simulated uranium‐containing wastewater (Tables S7, Supporting Information), achieving a remarkable removal efficiency of 95.74% (Figure 4g). Quantification via ICP‐MS revealed a uranium adsorption capacity of 56 mg g^−1^ in simulated wastewater, substantially exceeding uptake of competing ions (Figure 4h). Distribution coefficients (*K_d_*) further confirmed exceptional selectivity for UO_2_^2^⁺ (*K_d_* = 119.69 mL g^−1^), vastly outperforming values for co‐existing ions (Figure 4h, inset). Furthermore, the PXRD patterns of TP‐PYTO‐SA_20_ after immersion in 0.3 and 1 m HNO_3_ solutions for 12 h demonstrate that the material retains well‐defined diffraction peaks, further confirming its robust structural integrity even under acidic conditions (Figure S17, Supporting Information). These data compellingly demonstrate the outstanding potential of TP‐PYTO‐SA_20_ for practical radioactive uranium remediation in complex nuclear wastewater.

### 2.4 Mechanistic Analysis

**Figure 5:**
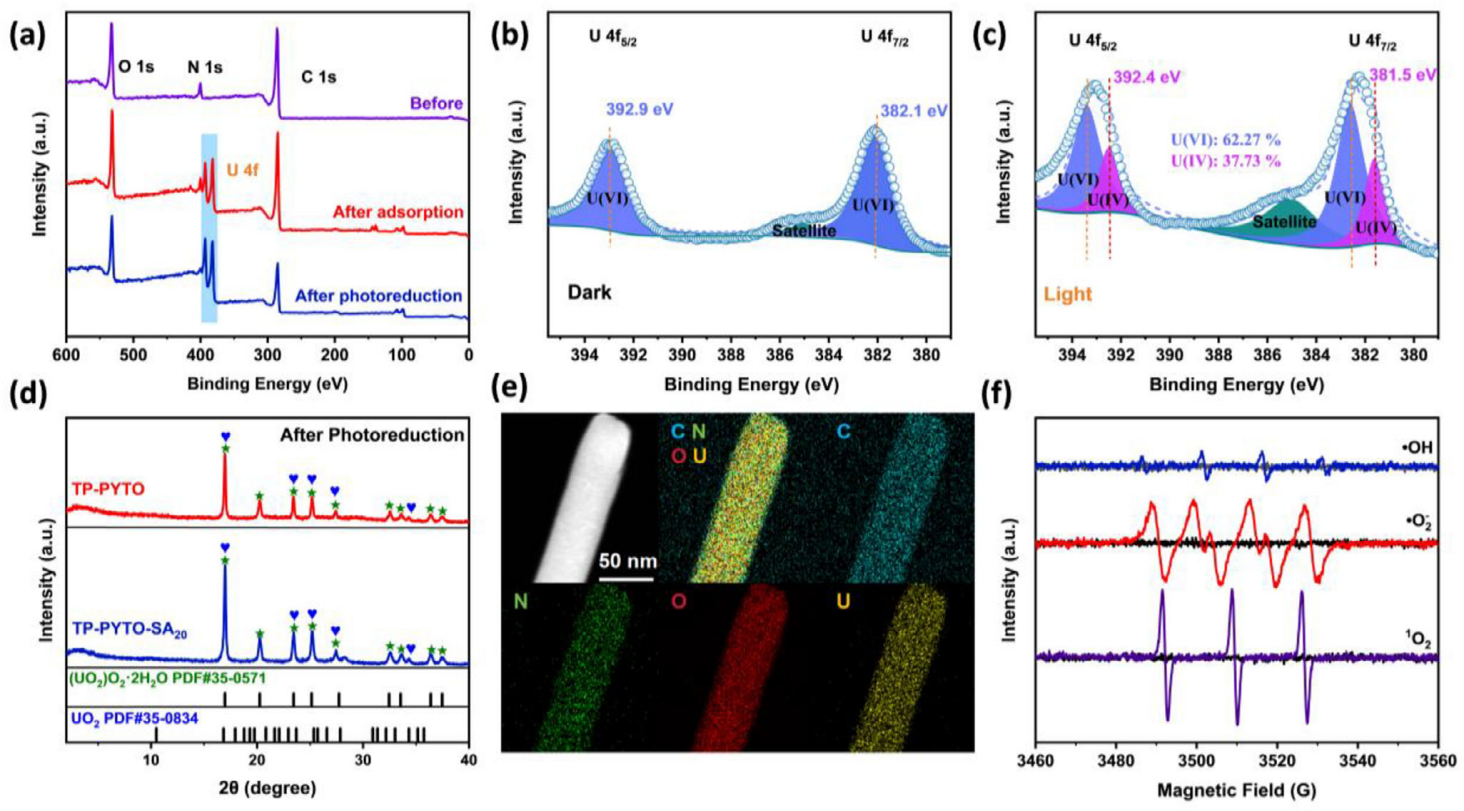
Interaction mechanism of TP‐PYTO‐SA_20_ with uranium. a) XPS measurement spectra of TP‐PYTO‐SA_20_ before and after uranium loading; b and c) U 4f high‐resolution spectra of uranium‐loaded TP‐PYTO‐SA_20_ under dark or light irradiation; d) The PXRD patterns after photoreduction of TP‐PYTO and TP‐PYTO‐SA_20_; e) TEM images and EDX elemental mapping images for TP‐PYTO‐SA_20_ after photoreduction; f) EPR spectra of •O_2_–DMPO, •OH–DMPO, and ^1^O_2_–TEMP adducts generated under light irradiation of TP‐PYTO‐SA_20_. Black curves represent samples kept in the dark; colored curves correspond to samples under light.

Given the exceptional uranium removal performance of TP‐PYTO‐SA_20_, we elucidated its underlying mechanism through X‐ray photoelectron spectroscopy (XPS) analysis before and after uranium binding. Under illumination, the intensified U 4f peaks following uranium adsorption contrasted markedly with spectra obtained under dark conditions (**Figure** **5**a). High‐resolution U 4f spectra revealed dominant peaks at 392.9 and 382.1 eV, corresponding to U 4f_5/2_ and U 4f_7/2_ of U(VI), confirming adsorption of hexavalent uranium in the absence of light (Figure 5b). Notably, after photoreduction, the U 4f spectrum of TP‐PYTO‐SA_20_ could be deconvoluted into four distinct components (Figure 5c). The emergence of new peaks at 392.4 and 381.5 eV, assigned to U 4f_5/2_ and U 4f_7/2_ of U(IV), provides direct spectroscopic evidence for the photocatalytic reduction of U(VI) to insoluble U(IV) species co‐existing on the material's surface.^[^ ^1^, ^62^ ^]^ Quantitative deconvolution of the U 4f XPS spectra revealed that 37.73% of surface uranium exists as U(IV), confirming substantial photocatalytic reduction. Furthermore, we determined the total uranium loading through complete digestion of the photocatalytically treated TP‐PYTO‐SA_20_ followed by ICP‐OES analysis, which yielded a value of 1066.48 mg g^−1^. Based on the XPS quantification, this corresponds to 402.38 mg g^−1^ of U(IV) and 664.10 mg g^−1^ of U(VI) species. This precise mass balance provides compelling evidence for the significant role of photocatalytic reduction in the uranium capture process, distinctly separating it from mere adsorption.

Complementary high‐resolution N 1s spectra of TP‐PYTO‐SA_20_ revealed characteristic binding energies at 399.4 and 400.3 eV, corresponding to the C═N and C─N bonds, respectively, within its structural linkages. Following uranium adsorption and photoreduction, the emergence of two new peaks at 400.4 and 400.8 eV in the N 1s spectrum was observed (Figure S18, Supporting Information). These peaks are unequivocally assigned to N‐U coordination bonds, indicating direct binding between uranyl ions (UO_2_^2^⁺) and nitrogen moieties within both the β‐ketoenamine and imine linkages of the framework. Concurrently, in the high‐resolution O 1s spectra, peaks associated with hydroxyl (O─H), ether (C─O), and carbonyl (C═O) functionalities exhibited distinct shifts toward higher binding energies post‐adsorption and photoreduction (Figure S19, Supporting Information).^[^ ^63^ ^]^ This systematic increase in binding energy provides direct spectroscopic evidence for the successful coordination of oxygen atoms with adsorbed UO_2_^2^⁺ species. Further characterization of uranium speciation was performed via PXRD analysis post‐adsorption and photocatalysis (Figure 5d; Figure S20, Supporting Information). Adsorbed uranium primarily existed as crystalline UO_2_(NO_3_)_2_(H_2_O)_6_ (PDF#72‐2205), UO_2_(NO_3_)_2_(H_2_O)_2_ (PDF#72‐2333) and UO_2_(NH_2_O)_2_(H_2_O)_4_ (PDF#70‐0659).^[^ ^64^ ^]^ Following photoreduction, distinct diffraction peaks emerged at 16.9°, 23.4°, 25.9°, and 32.6°, corresponding to characteristic reflections of metastudtite (UO_2_, PDF#35‐0834) and ((UO_2_)O_2_·2H_2_O, PDF#35‐0571), (All PDF cards are sourced from the ICDD database).^[^ ^62^ ^]^

This crystalline phase transformation confirms the photoreduction of U(VI) to U(IV) species. Mechanistically, conduction band (CB) electrons are consumed in reducing UO_2_^2^⁺ to UO_2_, while the formation of stable ((UO_2_)O_2_·2H_2_O) originates from the reaction of UO_2_⁺ with photogenerated •O^−^ superoxide radicals, the latter species arising from O_2_ activation by photoelectrons. Notably, sharper diffraction peaks corresponding to uranium compounds were observed in TP‐PYTO‐SA_20_ compared to other samples after both adsorption and photoreduction, indicating enhanced uranium enrichment within its framework. Based on the spectroscopic and diffraction evidence, the high U(VI) selectivity of TP‐PYTO‐SA_20_ can be explained by a two‐step mechanism. First, UO_2_^2^⁺ is captured from the solution through coordination with the carbonyl, hydroxyl, and imine nitrogen sites on the TP‐PYTO‐SA_20_. Second, under light irradiation, the adsorbed U(VI) is specifically and irreversibly reduced to insoluble U(IV) precipitates, as unambiguously identified by XPS and PXRD. This photocatalytic reduction step permanently immobilizes uranium and simultaneously regenerates the active sites for further capture. In contrast, the competing metal ions (e.g., Mg^2^⁺, Zn^2^⁺, Co^2^⁺) are not susceptible to such reduction under these conditions.

Complementary SEM, HR‐TEM elemental mapping, and energy‐dispersive X‐ray spectroscopy (EDX) analysis confirmed successful uranium deposition on TP‐PYTO‐SA_20_ nanorods (Figures 5e; S21, Supporting Information). These techniques corroborated the homogeneous distribution of carbon (C), oxygen (O), nitrogen (N), and uranium (U) across the COF surface, demonstrating uniform elemental dispersion within the material architecture. The HR‐TEM image of the solid nanoparticles adhered to TP‐PYTO‐SA_20_ clearly evidences the formation of UO_2_ (Figure S22, Supporting Information). Lattice fringes with a d‐spacing of ≈3.2 Å were observed, which can be readily indexed to the (111) plane of cubic UO_2_.^[^ ^65^ ^]^ A comparison of the FT‐IR spectra before and after adsorption reveals the emergence of a distinct characteristic peak at 928 cm^−1^, which is attributed to the asymmetric stretching vibration of the O═U═O bond, confirming the successful capture of uranium (Figure S23, Supporting Information).^[^ ^66^ ^]^ EPR results showed that, compared with the dark condition, intense signals of •O_2_^−^, •OH, and ^1^O_2_ were detected under light irradiation (Figure 5f). To further unequivocally identify the primary active species in the photocatalytic process, quenching experiments were conducted (Figure S24, Supporting Information). The U(VI) removal efficiency was severely suppressed upon the addition of scavengers for e^−^ and •O_2_^−^, whereas scavengers for hydroxyl radicals (•OH) and singlet oxygen (^1^O_2_) had a minimal impact. These results provide direct evidence that photogenerated electrons and •O_2_^−^ are the crucial species responsible for the photocatalytic reduction of U(VI), firmly supporting the proposed reaction pathway where electrons participate in direct reduction and •O_2_^−^ facilitates the formation of uranium peroxide precipitates. These experimental results provide crucial evidence for elucidating the mechanism of photocatalytic uranium reduction.

Based on the above results, the photocatalytic uranium removal mechanism of TP‐PYTO‐SA_20_ involves five consecutive steps: (1) Photoexcitation generates electron‐hole pairs (*e^−^* / *h⁺*); (2) Photogenerated electrons reduce O_2_ to form superoxide radicals (•O_2_^−^); (3‐4) Sequential reduction of UO_2_^2^⁺ yields intermediate UO_2_⁺ (U(V)) and ultimately precipitates UO_2_ (U(IV)); (5) The part of UO_2_⁺ (U(V)) with •O_2_^−^ and H_2_O to form crystalline metastudtite ((UO_2_)O_2_·2H_2_O).^[^ ^53^, ^67^ ^]^ This pathway aligns with the U(IV) species detected by XPS, PXRD, and TEM.

(1) $$\mathrm{COF}+h\nu \to {e^{-}}_{\left(\mathrm{CB}\right)}+{h^{+}}_{\left(\mathrm{VB}\right)}$$

(2) $$O_{2}+e^{-}\to \cdot O_{2}-$$

(3) $${\mathrm{UO}}_{2}^{2+}+e^{-}\to {\mathrm{UO}}_{2}^{+}$$

(4) $${\mathrm{UO}}_{2}^{+}+e^{-}\to UO_{2}\left(s\right)$$

(5) $${\mathrm{UO}}_{2}^{+}+\cdot O_{2}^{-}+2H_{2}O\to (UO_{2})O_{2}\cdot 2H_{2}O(s)$$

### 2.5 Density Functional Theory Calculations

**Figure 6:**
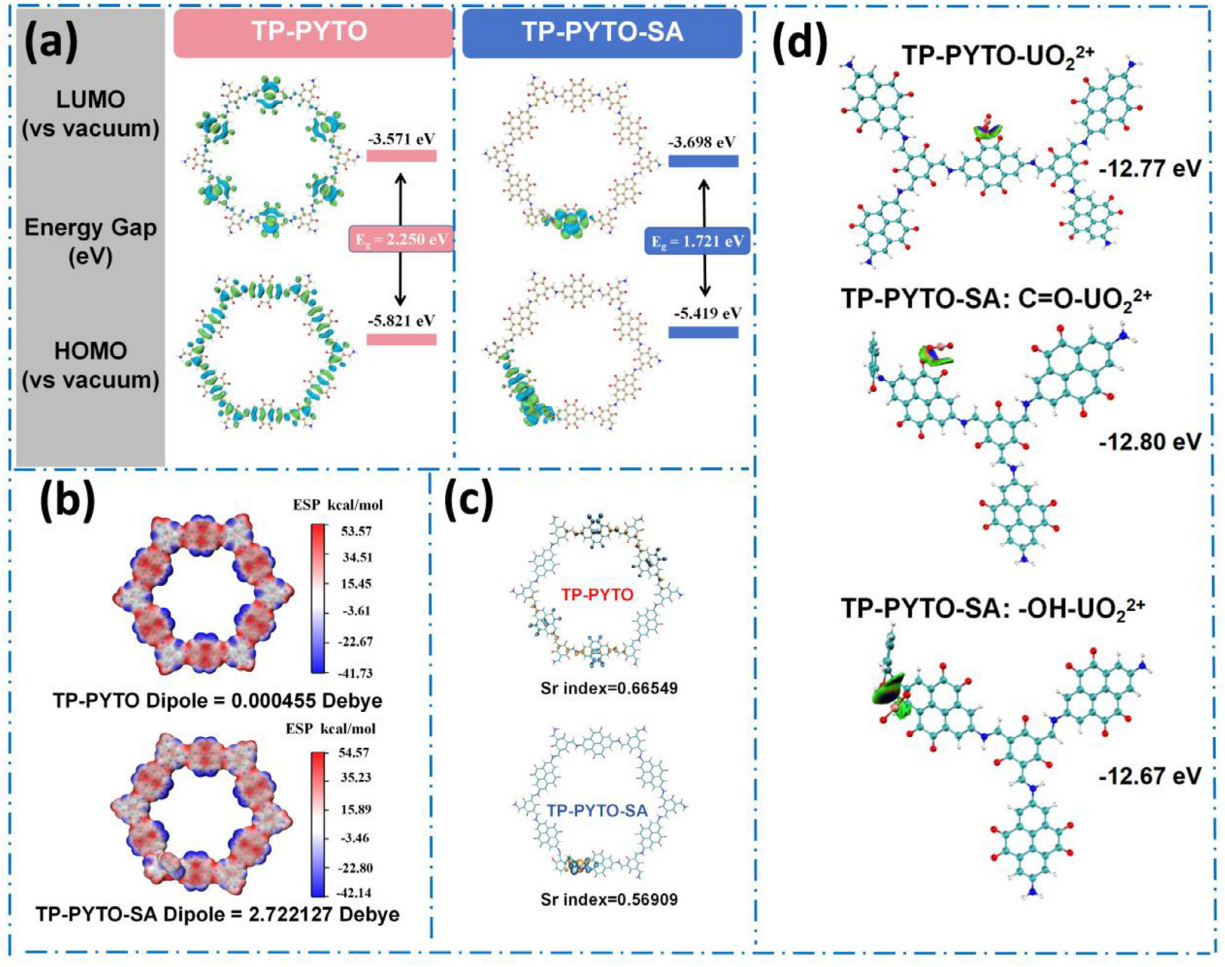
DFT calculations of TP‐PYTO and TP‐PYTO‐SA. a) HOMO–LUMO orbital distributions based on optimized ground‐state geometries; b) Electrostatic potential (ESP) maps; c) Electron–hole spatial distributions; d) Binding energies of TP‐PYTO and TP‐PYTO‐SA with uranium species.

Density functional theory (DFT) calculations revealed salicylaldehyde‐activated defect engineering mechanisms governing uranium extraction in pyrene‐based COF_S_. DFT calculations were performed using Gaussian 16 to elucidate the electronic structures and uranium binding mechanisms. DFT calculations analyzed the electronic structures of macrocyclic fragments in TP‐PYTO and TP‐PYTO‐SA (**Figure** **6**a). In TP‐PYTO, electrons in both the highest occupied molecular orbital (HOMO) and lowest unoccupied molecular orbital (LUMO) are delocalized across the entire framework. The introduction of defect structures significantly alters the electronic environment. TP‐PYTO‐SA with defects exhibits a donor‐acceptor (D‐A) conformation by transferring HOMO electrons to the electron‐donor unit, facilitating efficient electron transfer along the backbone. The emergence of this D‐A structure offers a fundamental explanation for the superior charge separation efficiency of TP‐PYTO‐SA_20_ seen in the photoluminescence and EPR measurements (Figure 3c–f). The facilitated electron transfer along the backbone rationalizes its lower charge transfer resistance and higher photocurrent density (Figure 3d,e). Frontier molecular orbitals, electrostatic potential distributions, electron‐hole density maps, and transition density matrices were analyzed using Multiwfn and visualized with VMD. Electrostatic potential (ESP) analysis reveals that TP‐PYTO‐SA possesses a substantially larger dipole moment (2.72 debye) compared to TP‐PYTO (4.5 × 10^−4^ debye), unequivocally demonstrating that defect incorporation generates a stronger BIEF (Figure 6b). ESP further visualizes the spatial arrangement of positive/negative charges in the COF rings: positive charges distribute above C atoms of the backbone, while negative charges localize above O atoms of carbonyl groups. The defect structures create localized D‐A configurations and concentrate positive charges within these segments, inducing charge polarization effects that promote charge separation.^[^ ^68^, ^69^ ^]^

Electron‐hole distribution was investigated (Figure 6c), TP‐PYTO‐SA exhibits a lower electron‐hole overlap integral (Sr index) than TP‐PYTO, confirming that defects promote directional electron movement and effectively suppress carrier recombination.^[^ ^70^ ^]^ The resonance electron‐donating effect of hydroxyl groups in TP‐PYTO‐SA further amplifies this phenomenon, leading to reduced Sr values. The reduced electron‐hole overlap provides a theoretical basis for the suppressed charge recombination observed in the steady‐state and time‐resolved PL spectra, explaining why TP‐PYTO‐SA_20_ maintains a higher population of free charges for photocatalysis. Analysis of the first five transition density matrices for both structures indicates that the incorporation of the SA markedly alters the electron‐hole overlap in the COF's excited states, leading to a more delocalized character of the electronic transitions (Figure S5, Supporting Information). To elucidate uranium coordination behavior, quantum chemical calculations were performed (Figure 6d). The U(VI) binding energy for TP‐PYTO is −12.77 eV, where uranium primarily coordinates with oxygen atoms of carbonyl groups. In TP‐PYTO‐SA, uranium simultaneously coordinates with both carbonyl oxygens (binding energy: −12.80 eV) and hydroxyl groups introduced by SA defects (binding energy: −12.67 eV). This synergistic coordination mechanism underpins TP‐PYTO‐SA's significantly enhanced uranium adsorption capacity. These binding energy calculations deliver a quantitative molecular‐level understanding of the experimental XPS results and the remarkable adsorption capacity. The presence of multiple, strong coordination sites in TP‐PYTO‐SA, as confirmed by DFT, directly explains its ability to effectively capture and concentrate uranium species, as seen in the elemental mapping (Figure 5e), and its high performance in both simulated wastewater and competitive ion experiments (Figure 4g,h).

## 3 Conclusion

In summary, the defect‐engineered pyrene‐based covalent organic frameworks have been successfully designed and synthesized via the strategic incorporation of salicylaldehyde as a monomer. The partial substitution not only introduces controlled structural defects but also provides phenolic hydroxyl groups, which synergistically enhance the framework's hydrophilicity and furnish additional coordination sites for uranyl ions. By optimally engineering the defect density to 20%, the electronic structure of the COF is optimized. Concurrently, the electron‐donating phenolic hydroxyl groups markedly enhance charge‐carrier separation, yielding a tailored bandgap of 1.70 eV and improved charge delocalization. Under photocatalytic conditions, U(VI) is reduced to U(IV), enabling continuous regeneration of active sites. Under standard AM 1.5G solar irradiation, TP‐PYTO‐SA_20_ achieves an excellent uranium adsorption capacity of 1151.65 mg g^−1^, representing a 38.82% enhancement over the pristine COF, and exhibits remarkable uranium removal capability in uranium‐containing wastewater containing complex competing ions. Moreover, the material demonstrates outstanding stability and recyclability, retaining over 90% of its performance after 10 consecutive cycles. Overall, this work achieves a synergistic enhancement of photocatalysis and hydrophilicity through targeted monomer substitution, providing a versatile approach for the design of multifunctional COF‐based adsorbents and offering a promising platform for sustainable uranium recovery and effective radioactive wastewater remediation. Future efforts will be directed toward addressing key challenges for practical application, including the process engineering compatibility, performance in complex ionic matrices, and long‐term operational stability.

## Conflict of Interest

The authors declare no conflict of interest.

## Supporting information




**Supporting File**: advs73223‐sup‐0001‐SuppMat.docx.

## Data Availability

The data that support the findings of this study are available from the corresponding author upon reasonable request.
