# Supplementary material for: Defect Engineering Activates Pyrene‐Based Covalent Organic Frameworks for Efficient Photocatalytic Uranium Capture
Source: Adv Sci (Weinh). 2025 Dec 23;13(11):e18948. doi: 10.1002/advs.202518948 (PMC12931169; doi:10.1002/advs.202518948)
Supplement: Supplementary file 1 — Supporting File: advs73223‐sup‐0001‐SuppMat.docx. [file ADVS-13-e18948-s001.docx]

**Supporting Information**

**Defect Engineering Activates Pyrene-Based Covalent Organic Frameworks for Efficient Photocatalytic Uranium Capture**

**Weikun Yao^1^, Guanbing Zhou^1^, Tao Liu^1^, Yihui Yuan^1^, Hui Wang*^1^, Ning Wang*^1^**

^1^ State Key Laboratory of Marine Resource Utilization in South China Sea

Hainan University, Haikou 570228, China

| * Corresponding author. *E-mail address*: | wangn02@foxmail.com (Ning Wang)  huiw0318@163.com (Hui Wang) |
| --- | --- |

1. **Experimental Section**

**Materials.** 2,7-diaminopyrene-4,5,9,10-tetraone (PYTO) were purchased from Jilin Chinese Academy of Sciences-Yanshen Technology Co., Ltd. 2,4,6-triformylphloroglucinol (TP) and salicylaldehyde (SA) were purchased from Aladdin Co., Ltd. Mesitylene and acetic acid, 1,4-dioxane, methanol, acetone, N, N-dimethylformamide, and tetrahydrofuran, NaOH, and HNO_3_ were purchased from Xilong Scientific Co., Ltd. The U(VI) stock solution (1000 mg L^−1^) was prepared by dissolving UO_2_(NO_3_)_2_·6H_2_O purchased from Beijing Warwick Chemical Co. Ltd. Ultrapure water was prepared from the Millipore system (18.2 MΩ cm). All the reagents purchased were analytical grade and were not further purified.

**Synthesis of TP-PYTO:** A 10 mL Schlenk tube was charged with 2,7-diaminopyrene-4,5,9,10-tetraone (PYTO, 0.075 mmol) and 2,4,6-triformylphloroglucinol (TP, 0.075 mmol). Mesitylene (0.75 mL) and 1,4-dioxane (0.75 mL) were added as cosolvents. The mixture was sonicated for 15 min followed by addition of acetic acid (6 M, 0.1 mL) as catalyst. After three freeze-pump-thaw degassing cycles, the vessel was flame-sealed under vacuum and heated at 120°C for 72 h. The resulting solid was collected by filtration, sequentially washed with N, N-dimethylformamide (DMF), tetrahydrofuran (THF), and methanol (3 × 10 mL each), and subjected to Soxhlet extraction with methanol for 12 h. Final drying under dynamic vacuum at 90°C for 12 h afforded TP-PYTO as a crystalline black solid (87% yield).

**Synthesis of TP-PYTO-SA:** Defect-engineered TP-PYTO-SAX (X=10, 20, and 30) materials were synthesized via a pre-condensation protocol. PYTO (0.075 mmol) and salicylaldehyde (molar equivalents corresponding to X% of total aldehyde functionality) were pre-reacted in mesitylene/1,4-dioxane (1:1 v/v, 1.5 mL total) at 70°C for 2 h under N₂. TP (adjusted stoichiometry to maintain 0.075 mmol total aldehydes) and acetic acid (6 M, 0.1 mL) were subsequently added. The mixture underwent identical sonication, degassing, and thermal cyclization (120°C, 72 h) procedures as TP-PYTO. Purification followed the same washing, Soxhlet extraction, and drying protocol, yielding phase-pure materials (82% average yield). This pre-reaction proved essential, as comparative PXRD analysis revealed that direct mixing produced amorphous byproducts, whereas the two-step protocol yielded phase-pure crystalline frameworks (Figure S26, Supporting Information).

**Characterization methods.** Powder X-ray diffraction (PXRD) data of the nanomaterials were collected on a Rigaku/Smart Lab 9KW (75 kV, 40 mA) using Cu Kα (λ=1.5418 Å) radiation. Fourier transform infrared spectroscopy (FT-IR, Frontier, Perki­nElmer) was used to analyze the functional groups of the materials. The morphology of the nanomaterials was imaged by a scanning electron microscopy and high-resolution transmission electron microscopy (SEM, JEM-2010, JEOL; HRTEM, FEI Talos F200X G2). X-ray photoelectron spectroscopy (XPS) spectra were performed on a Thermo VG Multilab 2000X with Al Kα irradiation. The nitrogen adsorption and desorption isotherms were measured at 77 K using a Micromeritics ASAP 2020M system. The samples were outgassed at 90 °C for 8 h before the measurements. Surface areas were calculated from the adsorption data using Brunauer-Emmett-Teller (BET) method. Water contact angle was calculated using contact angle measuring equipment (SL200B/K Kino, America). The surface energy and component composition were tested by OWEN two-liquid phase method. UV/Vis diffuse reflectance spectra (DRS) were recorded with a PE Lambda 900 UV/vis spectrophotometer at room temperature. Temperature-dependent photoluminescence (PL) measurements emission spectra and PL decay spectra were measured at room temperature using FLS 1000 spectrometer (Edinburgh Instruments, UK). Electron paramagnetic resonance (EPR) measurements in X-band (microwave frequency ≈ 9.83 GHz) were performed at 293 K by a Bruker E500-10/12 micro spectrometer. The concentration of uranium and competitive metal ions were determined by inductively coupled plasma optical emission spectrometer (ICPMS7200, ThermoFisher, USA) and ultraviolet–visible absorption spectrophotometer (UV1800PC, Shanghai Jinghua Instruments Co., Ltd., China).

**Apparent surface energy.** Water (2 μL) contact angle and diiodomethane (2 μL) contact angle was calculated using contact angle measuring equipment (SL200B/K Kino, America), respectively. The surface energy and component composition were tested by OWEN two-liquid phase method. In the equation (1), $\gamma_{L}$ are the surface tension (Surface Free Energy) of the solid, $\gamma_{S}^{d}$ and $\gamma_{S}^{p}$ are the non-polar and polar Component of the Solid's Surface Free Energy. $\gamma_{L}^{d}$ and $\gamma_{L}^{p}$ are the non-polar and polar Component of the Liquid's Surface Tension. $\theta$ is the contact angle of the liquid on the solid surface. ^[1]^

| $\gamma_{L}\left( 1+\cos\theta\right)=2\left( \gamma_{S}^{d}\cdot\gamma_{L}^{d} \right)^{1/2}+2\left( \gamma_{S}^{p}\cdot\gamma_{L}^{p} \right)^{1/2}$ | (1) |
| --- | --- |

**Photoelectric performance test.** The Mott-Schottky plots were measured at the frequencies of 500, 1000, and 1500 Hz. The Mott-Schottky measurements were performed on a CHI 660B electrochemical workstation in 0.1 M Na_2_SO_4_ electrolyte with Ag/AgCl electrode as the reference electrode and a Pt mesh as the counter electrode. ^[2]^ Steady-state photoluminescence (PL) emission spectra and PL decay spectra were measured at room temperature using FLS 1000 spectrometer (Edinburgh Instruments, UK). ^[3]^ Photoelectrochemical measurements were carried out on CHI 660B electrochemical workstation, with a conventional three electrodes cell (Indium-tin oxide (ITO) glasses as work electrode, a platinum mesh as the counter electrode and an Ag/AgCl electrode as reference electrode) at a particular voltage. The photocurrent responses were conducted with a CHI 660B workstation, with the working electrodes irradiated from the front side. The light was generated by a 300 W xenon lamp (PLS-SXE300D) with a light density of 1 kW m^-2^ at room temperature with the light wavelength from 300 nm to 2500 nm. Electrochemical impedance spectroscopy (EIS) measurements were performed in dark at open-circuit voltage in the frequencies range of 0.01 Hz to 10^5^ Hz. ^[4]^

**The test conditions for EPR.** EPR measurements in X-band (microwave frequency ≈ 9.83 GHz) were performed at 293 K by a Bruker E500-10/12 micro spectrometer. The samples were illuminated by a 300 W Xe lamp with 420 nm cut-off filter (LOT Oriel). All the samples were measured under the same conditions (microeave power: 0.63 mW, modedulation frequency: 65 KHz, modulation amplitude: 1 G, sweep time 40 S). g values have been caculated from the resonance fied B_0_ and the resonance frequency ν using the resonance condition *hν* = gβB_0_. ^[5]^

**Determination of Optimal pH.** In order to determine the optimal pH of uranium uptake, 20 mL 1000 mg L^-1^ uranium solution was diluted in deionized water to prepare 200 mg L^-1^ uranium solution. The pH of the 200 mg L^-1^ uranium solution were adjusted to 1.0, 2.0, 3.0, 4.0, 5.0 by 0.3 M NaOH and 0.3 M HNO_3_ solution, respectively. ^[6]^ Then, 10 mg COF powder was transferred into 100 mL of 200 mg L^-1^ uranium solution, and the uranium uptake experiments were carried out at 25 °C and at a shaking of 500 rpm for 10 h under standard AM 1.5G solar irradiation (Xenon-Lamp, 1 kW m^-2^). Throughout the adsorption process, the uranium concentration before and after uptake was determined by Arsenazo III method. In detail, 3 mL deionized water, 0.8 mL HCl (0.1 M), 0.2 mL Arsenazo III (0.1 mM), and 1 mL filtered uranium solution were mixed to obtain the test sample. The uranium concentration of the test samples were determined by UV-Vis spectrophotomemter at the wavelength of 652 nm and the uranium adsorption capacity of adsorbent was consequently calculated by Eq. (2).

| $q_{t}=\frac{(C_{0}-C_{t})\times V}{m}$ | (2) |
| --- | --- |

where $C_{0}$ (mg L^−1^) is the initial uranium concentration before uptake, $q_{t}$ (mg g^-1^) is the uranium adsorption amount after a contact time of t, $V$ (L) is the volume of uranium spiked solution, $C_{t}$ (mg L^−1^) is the instant uranium concentration at time t, and $m$ is the weight of the COF powder.

**Photocatalytic uranium removal kinetics.** The COF powder (10 mg) was transferred into 100 mL of 50 mg L^-1^ and pH = 5.0 uranium solution. After ultrasonic treatment for 15 min, the mixture was vigorously stirred under standard AM 1.5G solar irradiation (Xenon-Lamp, 1 kW m^-2^). Multiple samples were taken during the three-hour adsorption test. The percentage removal of uranium concentration was calculated by Eq. (3).

| $R\%=\frac{C_{0}-C_{e}}{C_{0}}\times100\%$ | (3) |
| --- | --- |

Where $C_{0}$ and $C_{e}$ (mg L^-1^) are the initial concentration and the final equilibrium concentration of U(VI), respectively.

**Photocatalytic uranium adsorption kinetics test.** The COF powder (10 mg) was transferred into 100 mL of 200 mg L^-1^ and pH = 5.0 uranium solution. After ultrasonic treatment for 15 min, the mixture was vigorously stirred under standard AM 1.5G solar irradiation (Xenon-Lamp, 1 kW m^-2^). Multiple samples were taken during the ten-hour adsorption test. The experimental data was fitted using Pseudo-first-order and Pseudo-second-order kinetic model, Eq. (4) and Eq. (5).

| $\ln(q_{e}-q_{t})=\ln\text{q}_{\text{e}}-\text{k}_{\text{1}}t$ | (4) |
| --- | --- |
| $\frac{t}{q_{t}}=\frac{1}{k_{2}q_{e}^{2}}+\frac{t}{q_{e}}$ | (5) |

where $q_{t}$ and $q_{e}$ (mg g^-1^) represent the removal amount at time and at equilibrium *t* (min), respectively, $k_{1}$ and$k_{2}$ are rate constants of adsorption.

**Photocatalytic uranium adsorption isotherms test.** The adsorption isotherms were determined by testing the uranium adsorption capacities in uranyl nitrate aqueous solution with uranium concentration ranging from 10 mg L^-1^ to 400 mg L^-1^, prepared by diluting 1000 ppm uranium solution with deionized water. COF powder of 10 mg adsorbent was dispersed in 100 mL uranium solution (pH 5.0). The adsorption isotherm was fitted with the Langmuir adsorption model with Eq. (6).

| $\frac{C_{e}}{q_{e}}=\frac{C_{e}}{q_{m}}+\frac{1}{k_{3}q_{m}}$ | (6) |
| --- | --- |

where $C_{e}$ (mg L^-1^) represents the uranium concentration at equilibrium, $q_{e}$ (mg g^-1^) and $q_{m}$ (mg g^-1^) are the uranium adsorption capacity at equilibrium and the maximum adsorption capacity, respectively, and $k_{3}$ (g mg^-1^ min^-1^) is equilibrium constants related to the binding strength.

**Ion selective test.** The COF powder (20 mg) was added to 100 mL mixed solution and stirred for 10 h under standard AM 1.5G solar irradiation (Xenon-Lamp, 1 kW m^-2^). The treated solution was filtered through a 0.22 μm membrane filter, and the filtrate was analyzed by ICP-MS to determine the residual ions concentration. The concentration ratio of U(VI) (20 mg L^-1^) and Competitive ions was prepared at 1:3 to carry out the photocatalytic experiment. The percentage removal of uranium concentration was calculated as Eq. (3).

**Test for uranium removal in** **simulated** **uranium-containing wastewater.** The uranium-containing wastewater with initial uranium concentration of 18.9 mg L^-1^ was used to determine the uranium removal ability of TP-PYTO-SA_20_. Herein, the concentrations of U, Mg, Zn, Sr, Ba, Co, Ni, Cu, Fe, and Cd were listed in Table S5 (Supporting Information). 20 mg TP-PYTO-SA_20_ was soaked into 50 mL uranium-containing wastewater for ions adsorption at 25 °C under standard AM 1.5G solar irradiation (Xenon-Lamp, 1 kW m^-2^). After adsorption for 12 h, the adsorption capabilities for different ions were determined by ICP-MS to evaluate the adsorption selectivity of the TP-PYTO-SA_20_ for uranium and other ions. The distribution coefficients of each ion in the simulated uranium-containing wastewater were calculated by Eq. (7).

| $K_{d}=\frac{Q_{e}}{C_{e}}=\frac{C_{0}-C_{e}}{C_{e}}\times\frac{V}{m}$ | (7) |
| --- | --- |

Where $K_{d}$ (mL g^-1^) is the distribution coefficient, $C_{0}$ and $C_{e}$ (mg L^-1^) are the initial concentration and the final equilibrium concentration of U(VI), V (L) is the volume of uranium spiked solution, and m is the weight of the COF powder, respectively.

**Reusability assay.** After one run of photocatalytic process, the resulting suspension was filtered. The solid residue was immersed and washed three times with 100 mL of a 0.1 M HNO₃ solution, followed by three rinses with 100 mL of ultrapure water. This washing process was repeated until the supernatant became clear. The resulting powder was collected, dried under vacuum, and then employed in the subsequent catalytic cycle experiment. ^[7]^ **Theoretical Calculation**

For structures without uranium were initially optimized using the B3LYP functional in combination with the 6-311G basis set. For uranium-containing systems, the MWB60 pseudopotential basis set was employed. Dispersion corrections were applied to all calculations to enhance accuracy. ^[8]^ Subsequent structural optimizations and thermodynamic frequency analyses for adsorption energy calculations were carried out using the PBE0 functional with the 6-311G basis set for carbon, oxygen, hydrogen, and nitrogen atoms, while the PBE0 functional combined with the MWB60 pseudopotential basis set was used for uranium atoms. ^[9]^ Excited-state structures were further re-optimized using the CAM-B3LYP functional along with the 6-311G basis set. Frontier molecular orbital data, electrostatic potential distributions, electron-hole density maps, and the first five excited-state electron density matrices derived from DFT calculations were analyzed using Multiwfn software (Multiwfn_3.8_dev_bin_Win64). Visualization of frontier orbitals, electrostatic potential maps, and electron-hole distributions was performed using Multiwfn in conjunction with VMD (Version 1.9.3). ^[10]^

1. **Characterization of Photocatalysts**

**
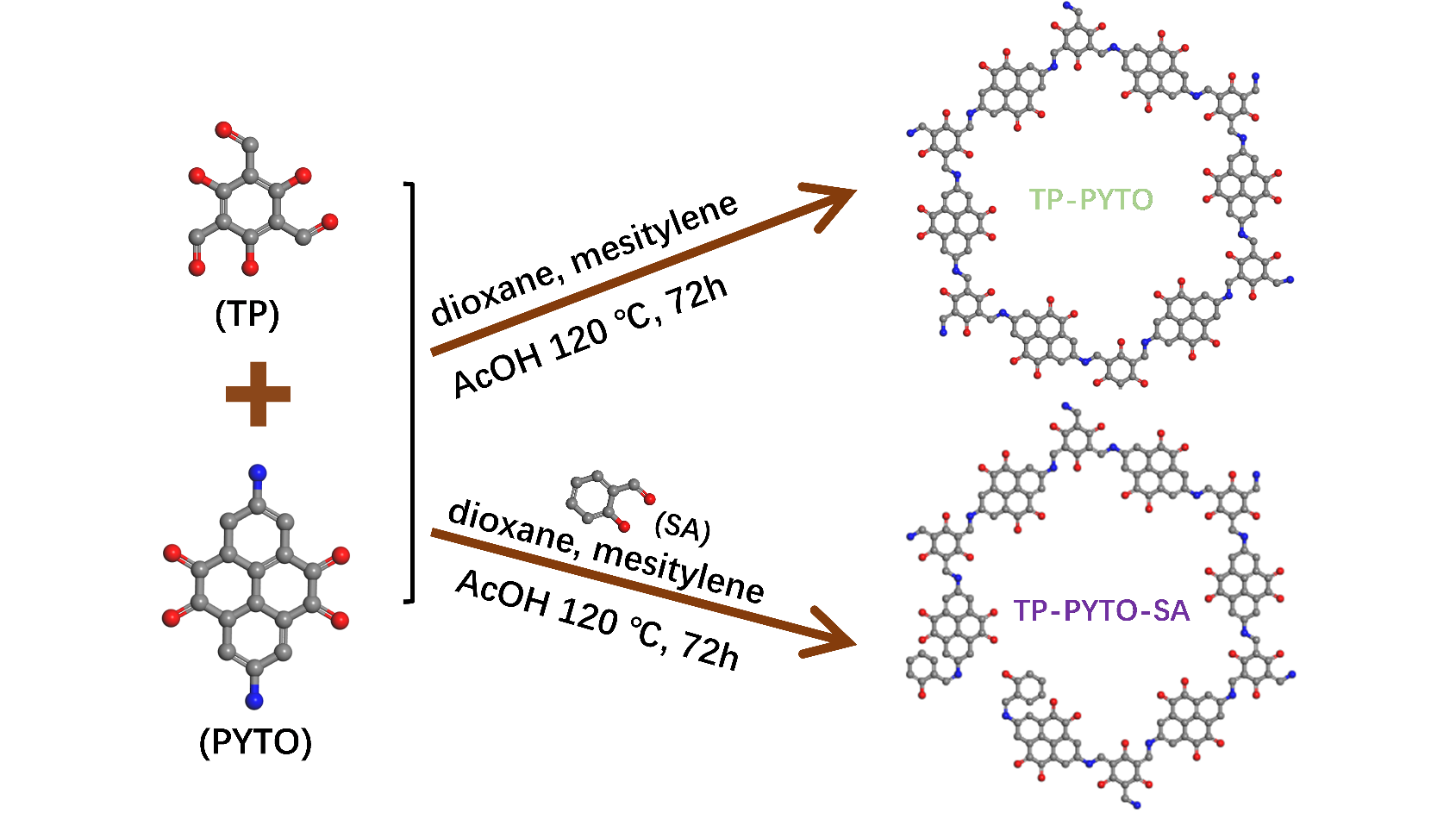
**

**Figure S1.** Schematic diagram of the synthesis of TP-PYTO and TP-PYTO-SA.


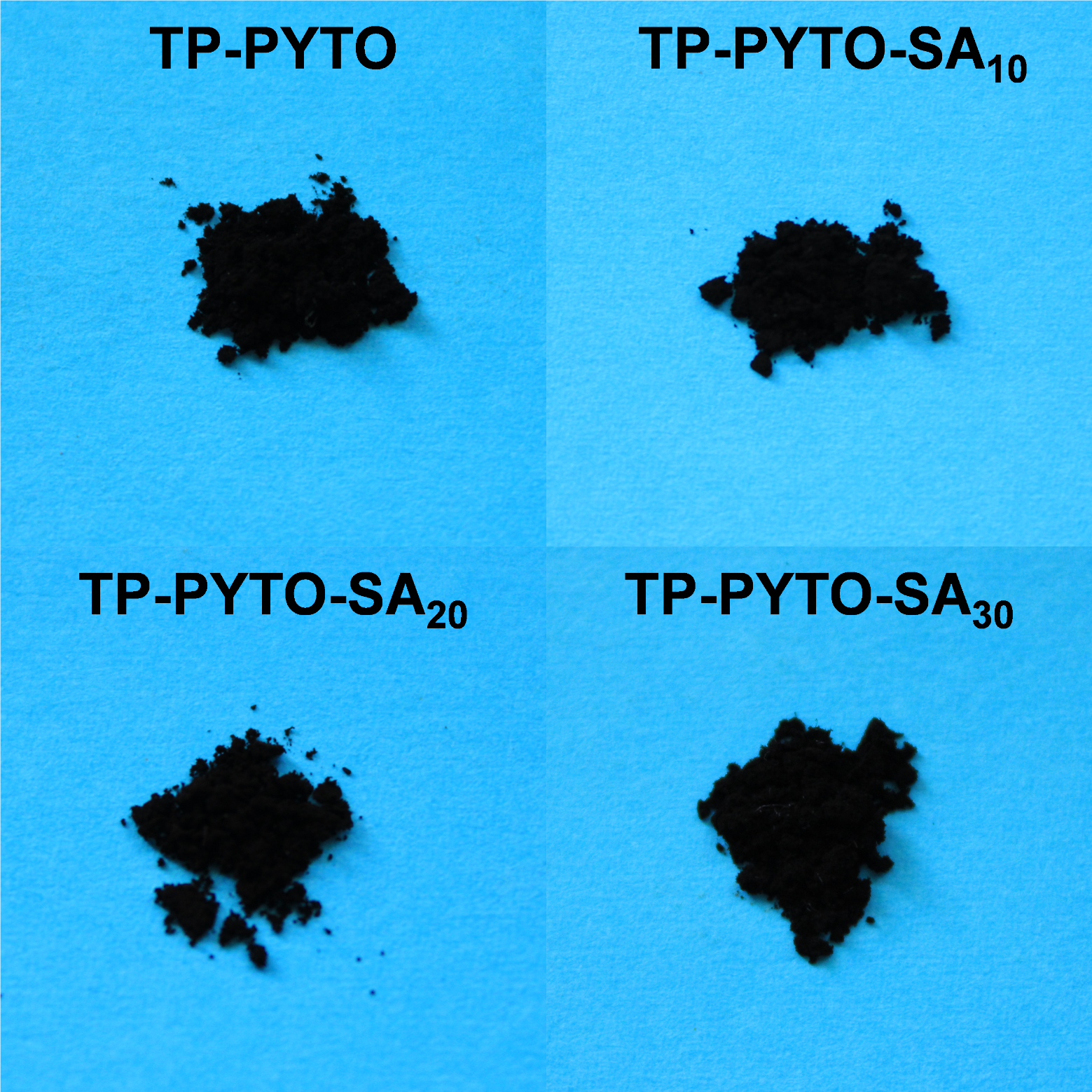


**Figure S2.** Digital images of TP-PYTO, TP-PYTO-SA_10_, TP-PYTO-SA_20_, and TP-PYTO-SA_30_.

**
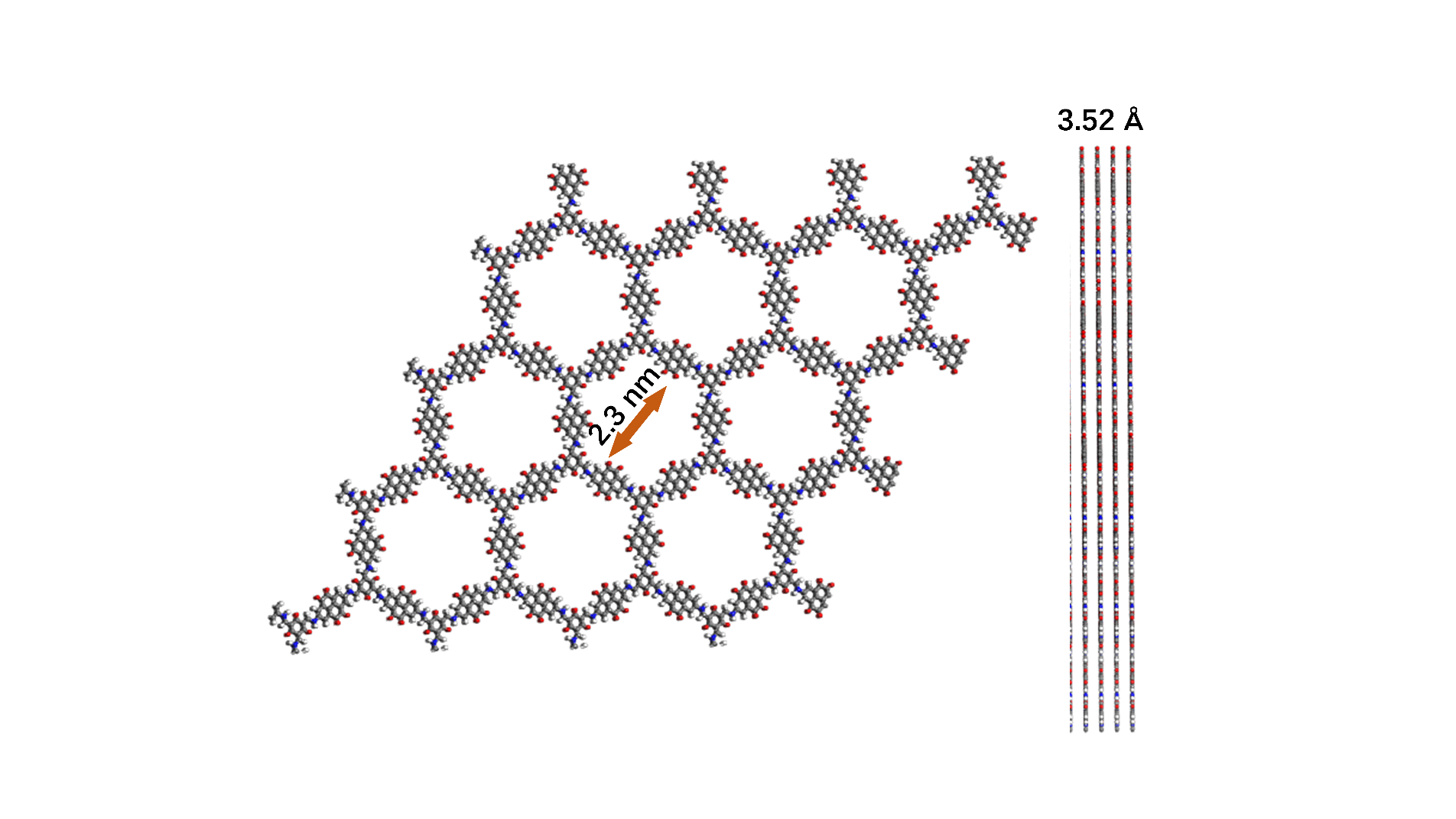
**

**Figure S3.** TP-PYTO structure simulated using Materials Studio.


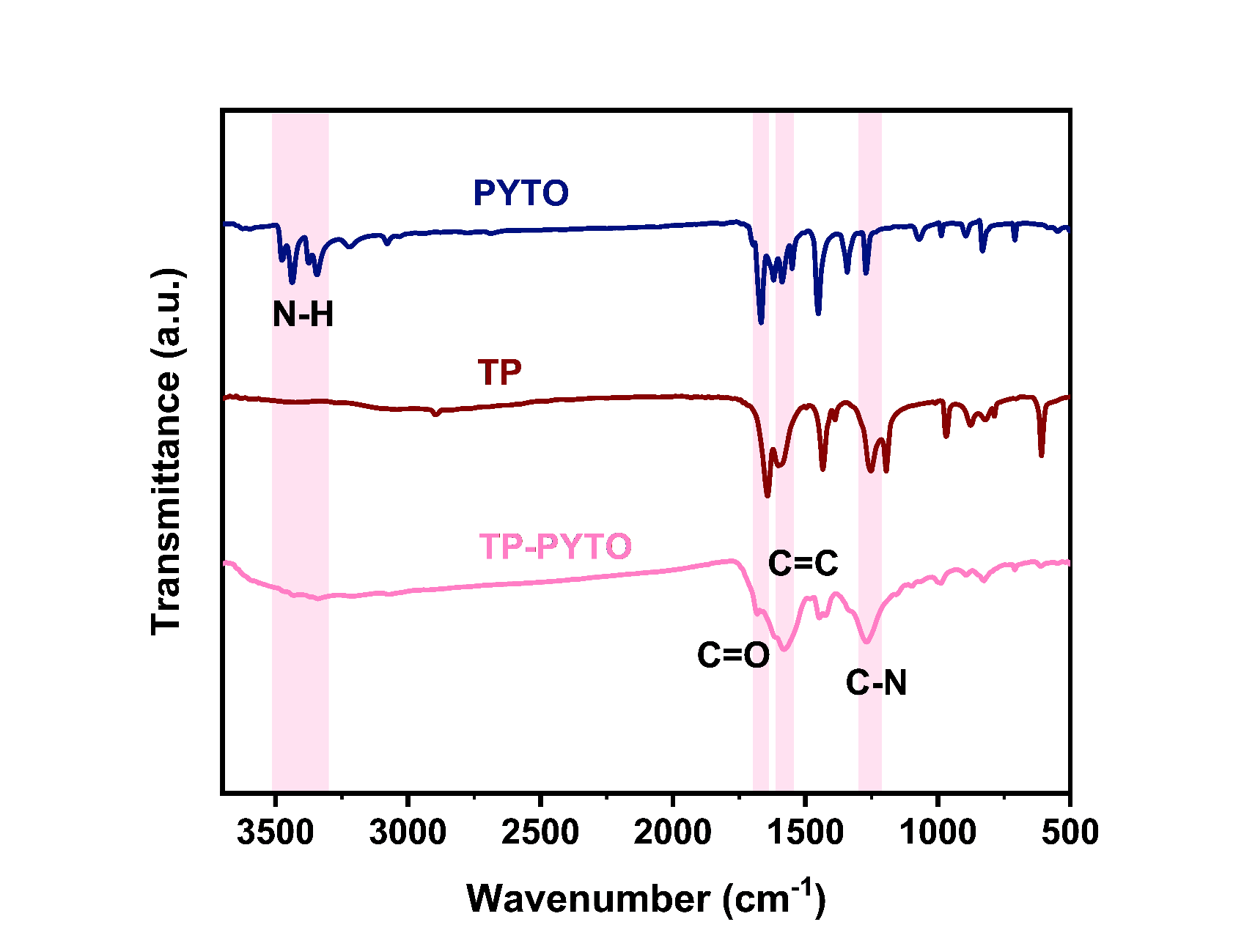


**Figure S4.** FT-IR spectra of PYTO, TP, and TP-PYTO.

**
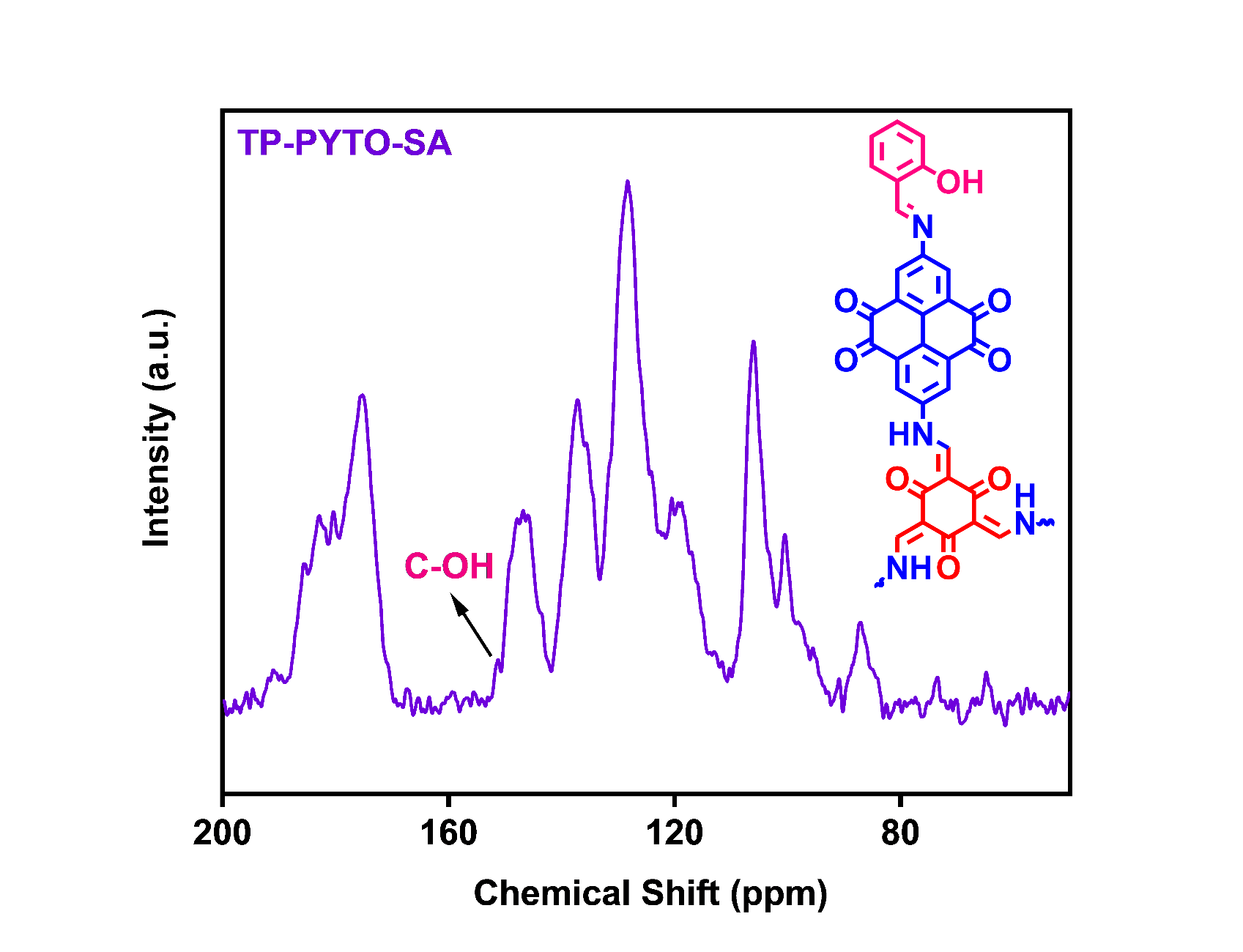
**

**Figure S5.** Solid-state ^13^C NMR spectra of TP-PYTO-SA.


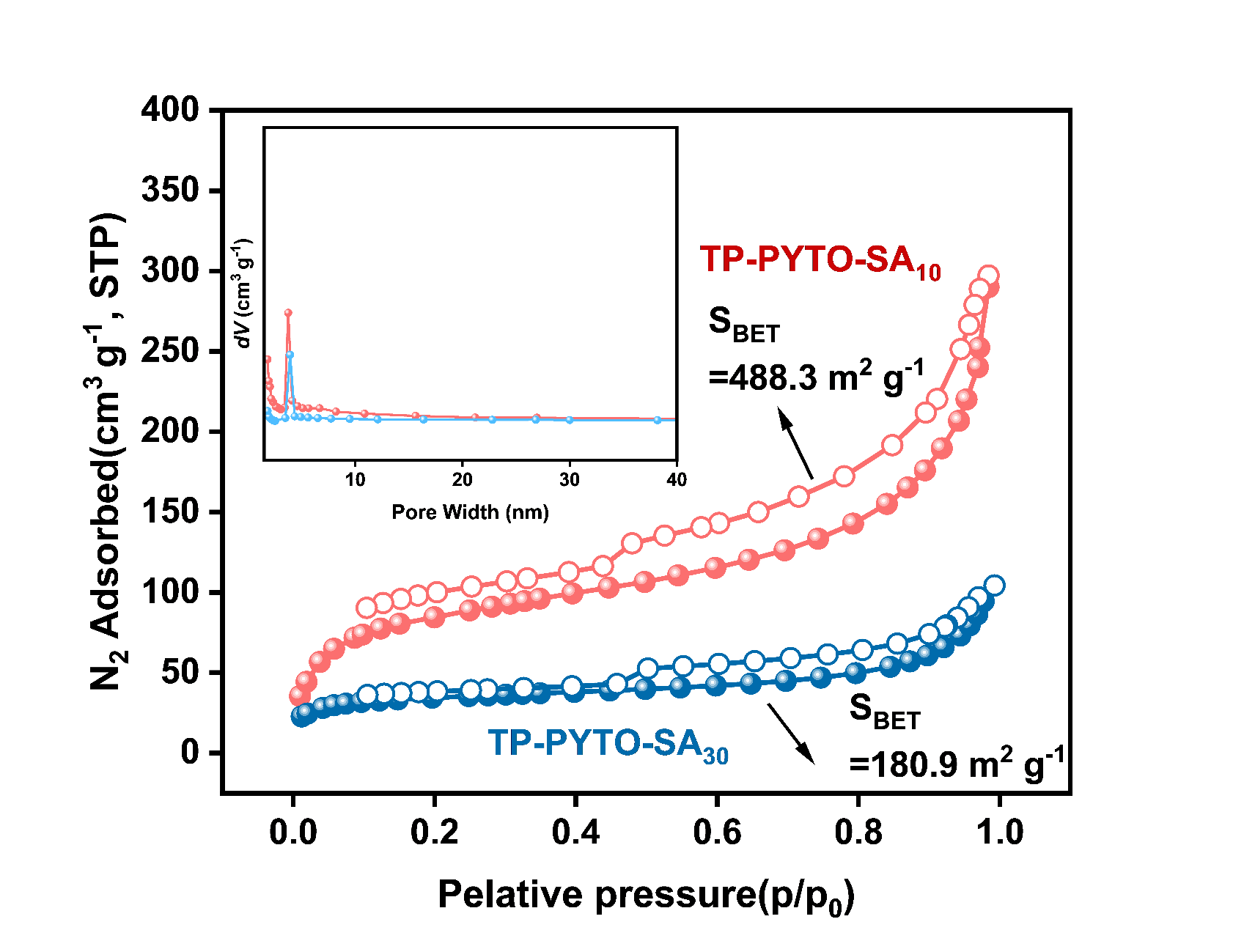


**Figure S6.** N_2_ adsorption and desorption isotherms of TP-PYTO-SA_10_ and TP-PYTO-SA_30_.

**
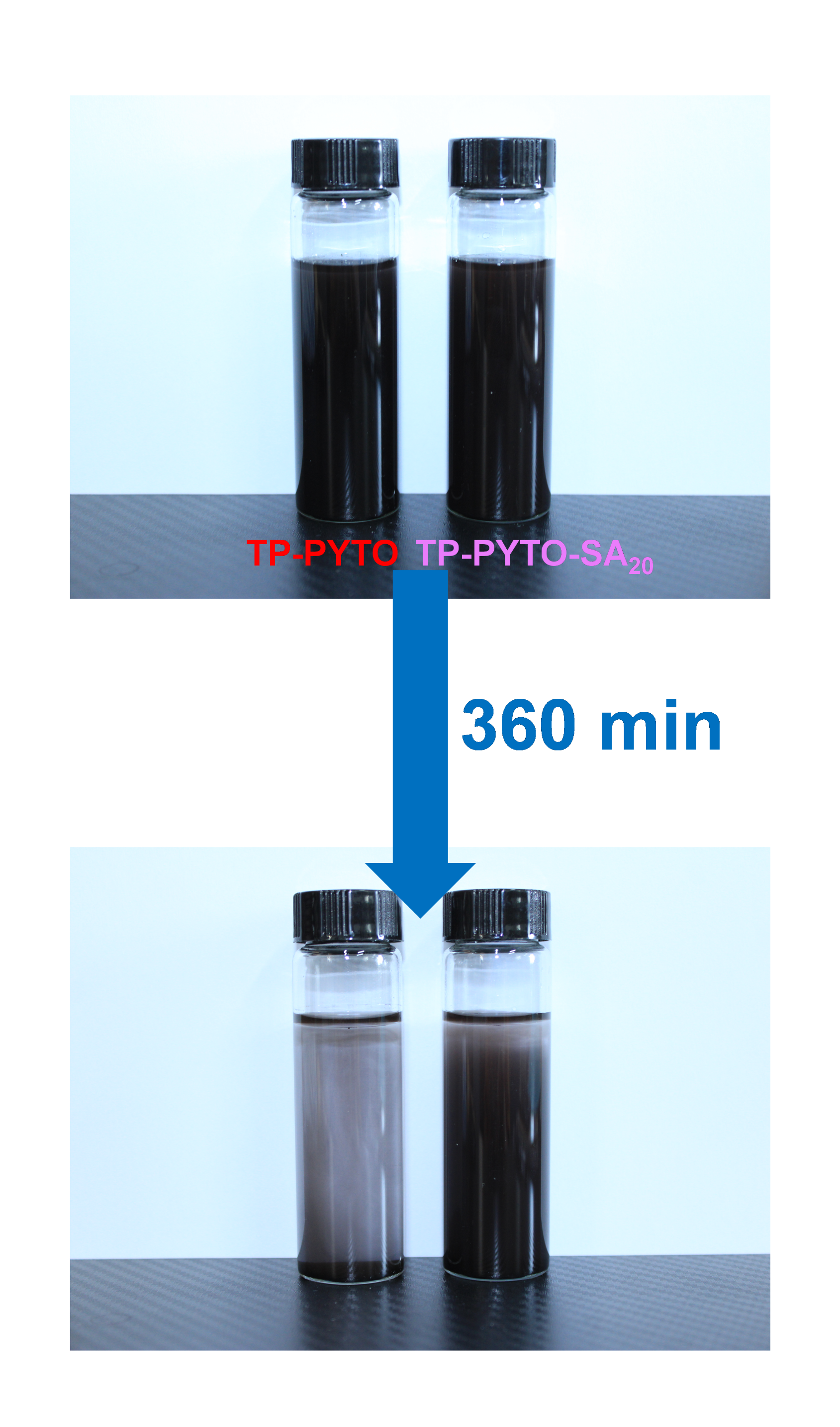
**

**Figure S7.** Digital image of COF powder uniformly dispersed in aqueous solution and after standing for 360 minutes.

**
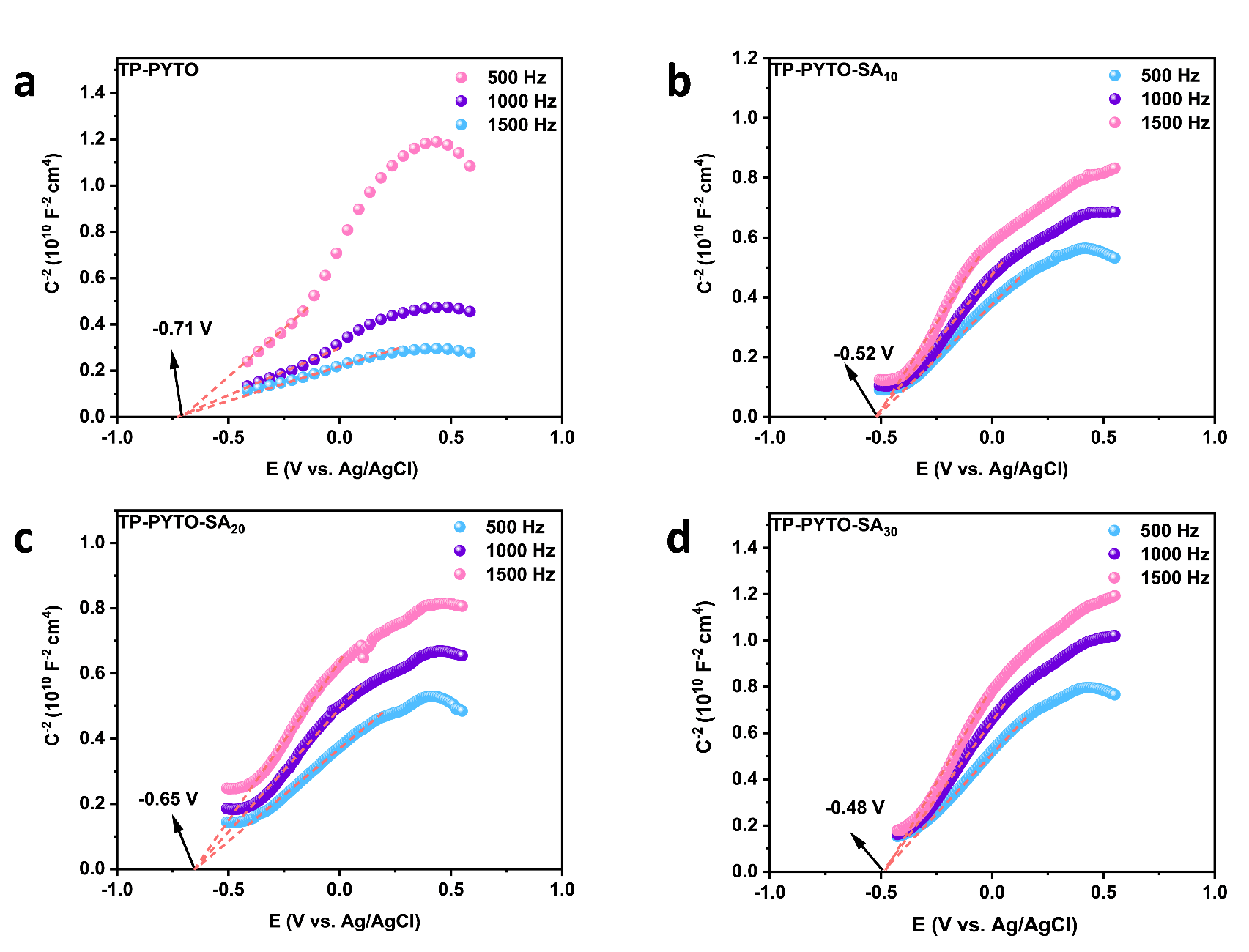
**

**Figure S8.** Mott-Schottky plots of a) TP-PYTO, b) TP-PYTO-SA_10_, c) TP-PYTO-SA_20_, and d) TP-PYTO-SA_30_.


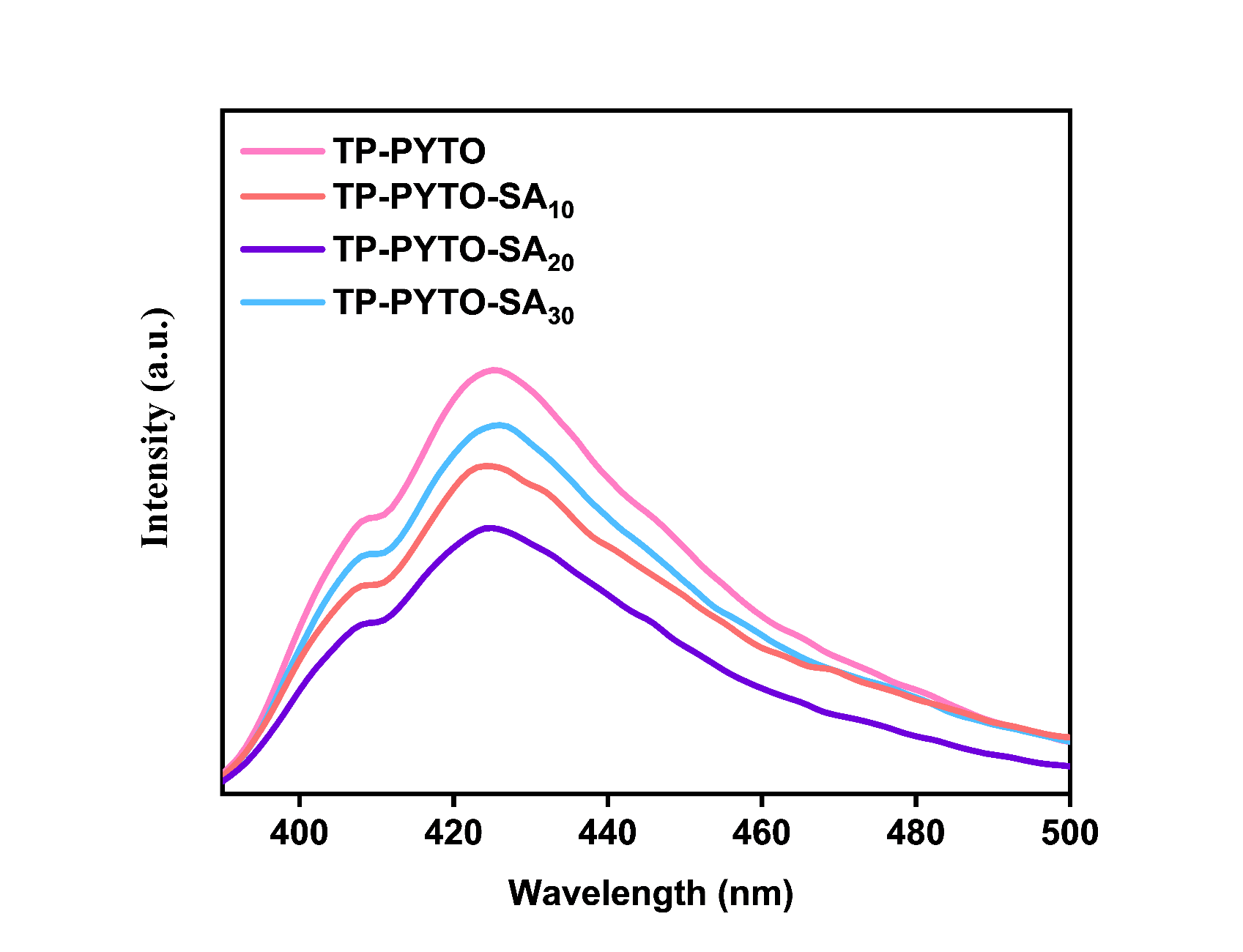


**Figure S9.** Static resolved PL emission spectroscopy of TP-PYTO, TP-PYTO-SA_10_, TP-PYTO-SA_20_, and TP-PYTO-SA_30_.


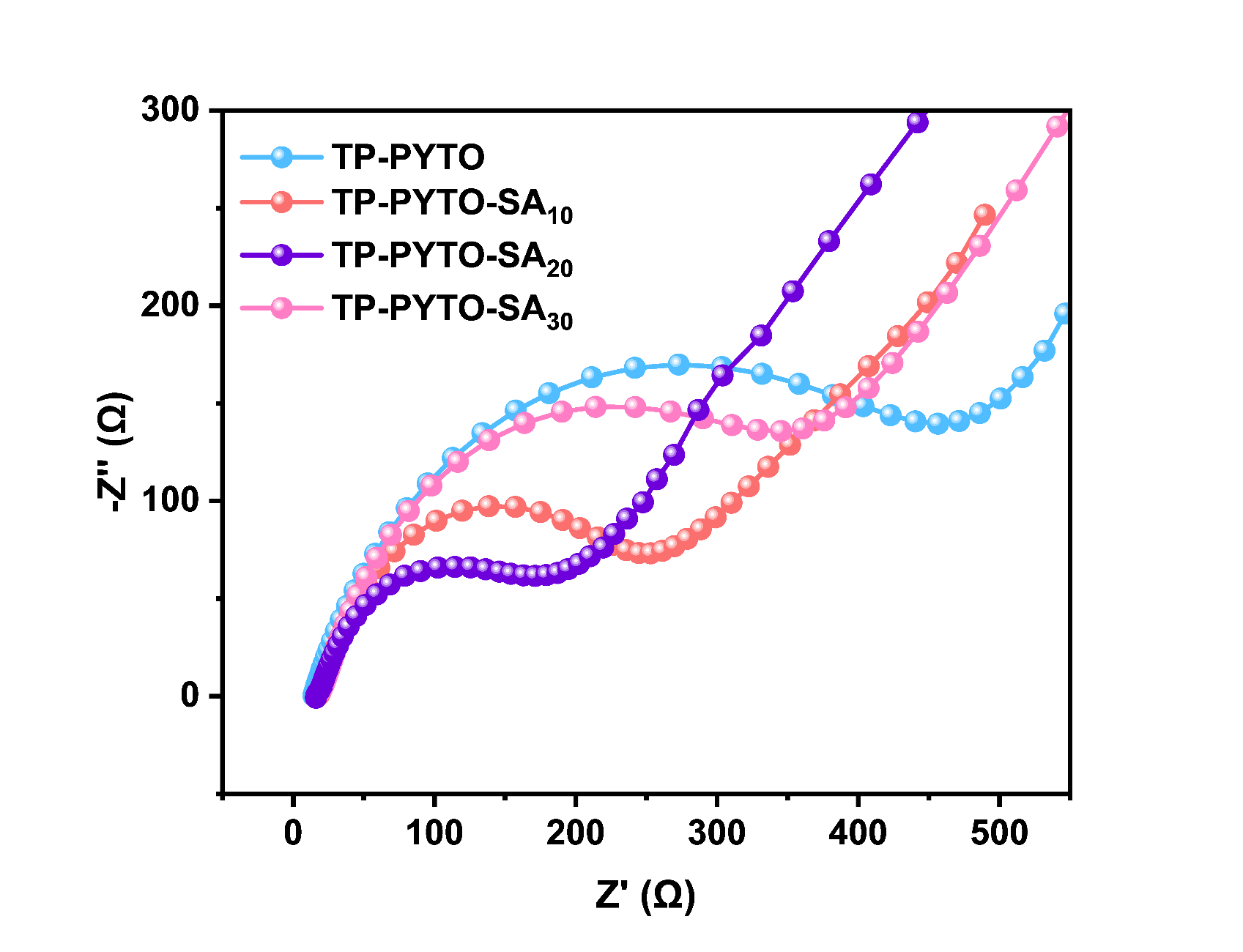


**Figure S10.** EIS spectra fitted using Zview.


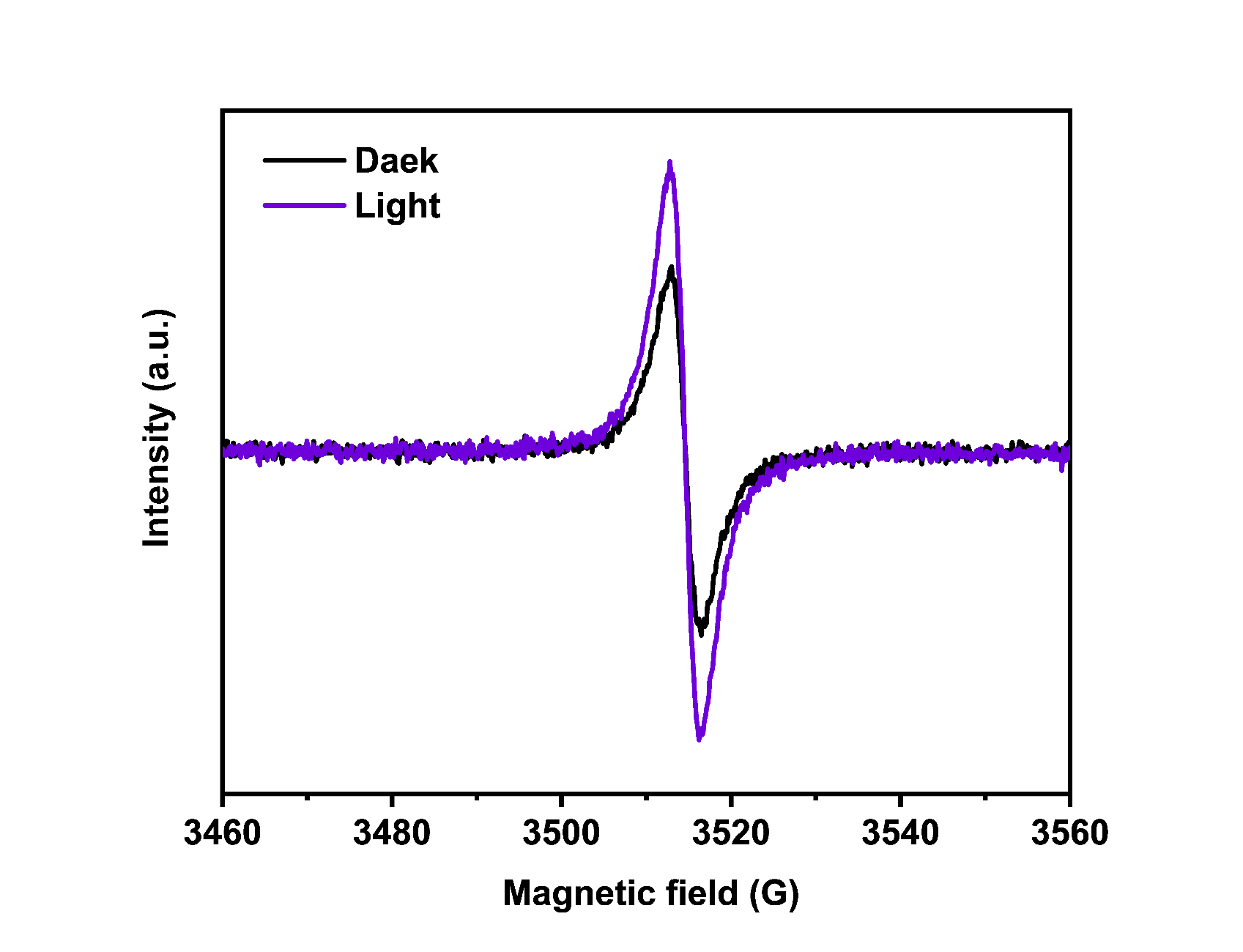


**Figure S11.** EPR spectra of TP-PYTO-SA_20_ under dark and light.

**
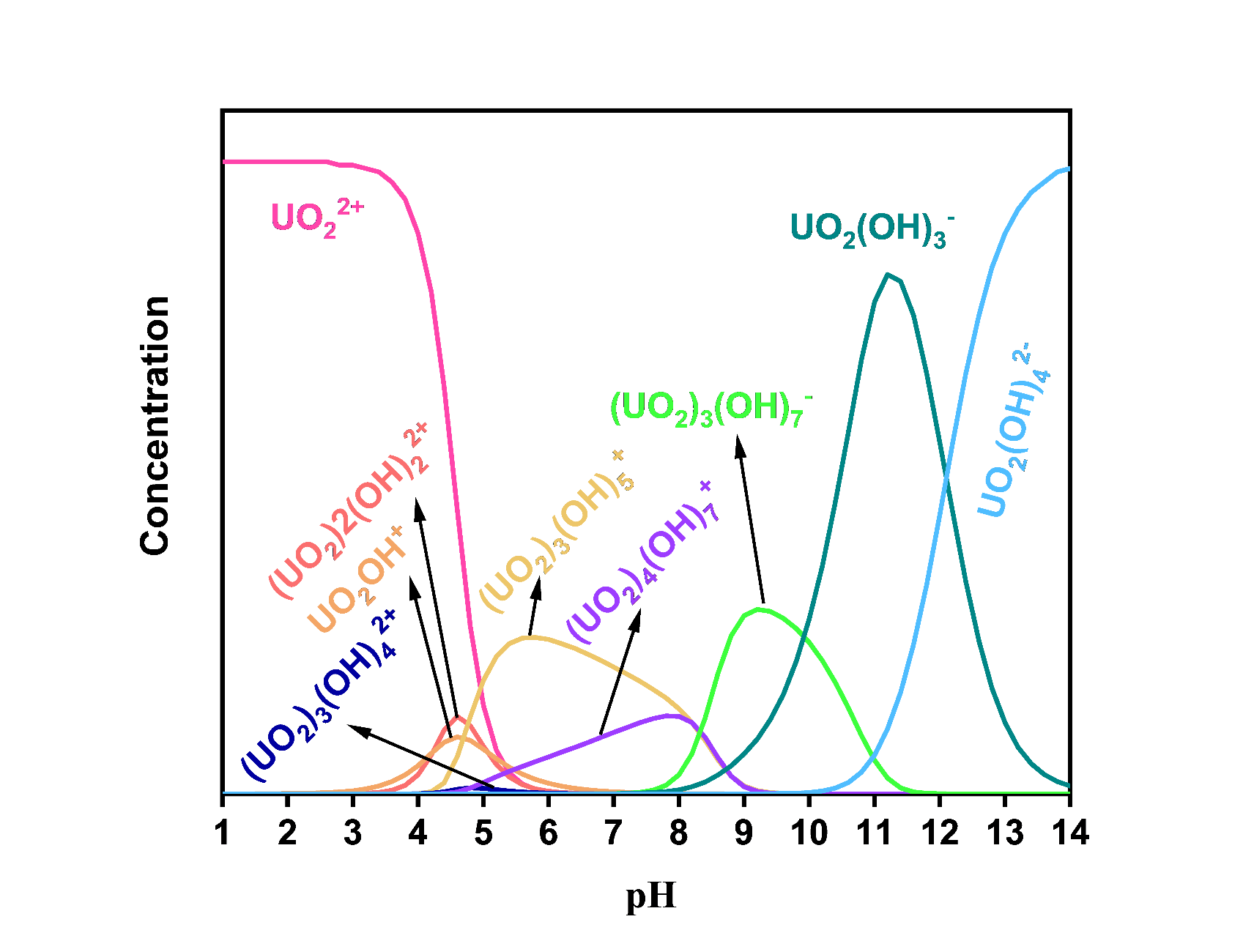
**

**Figure S12.** Uranium forms with different pH.


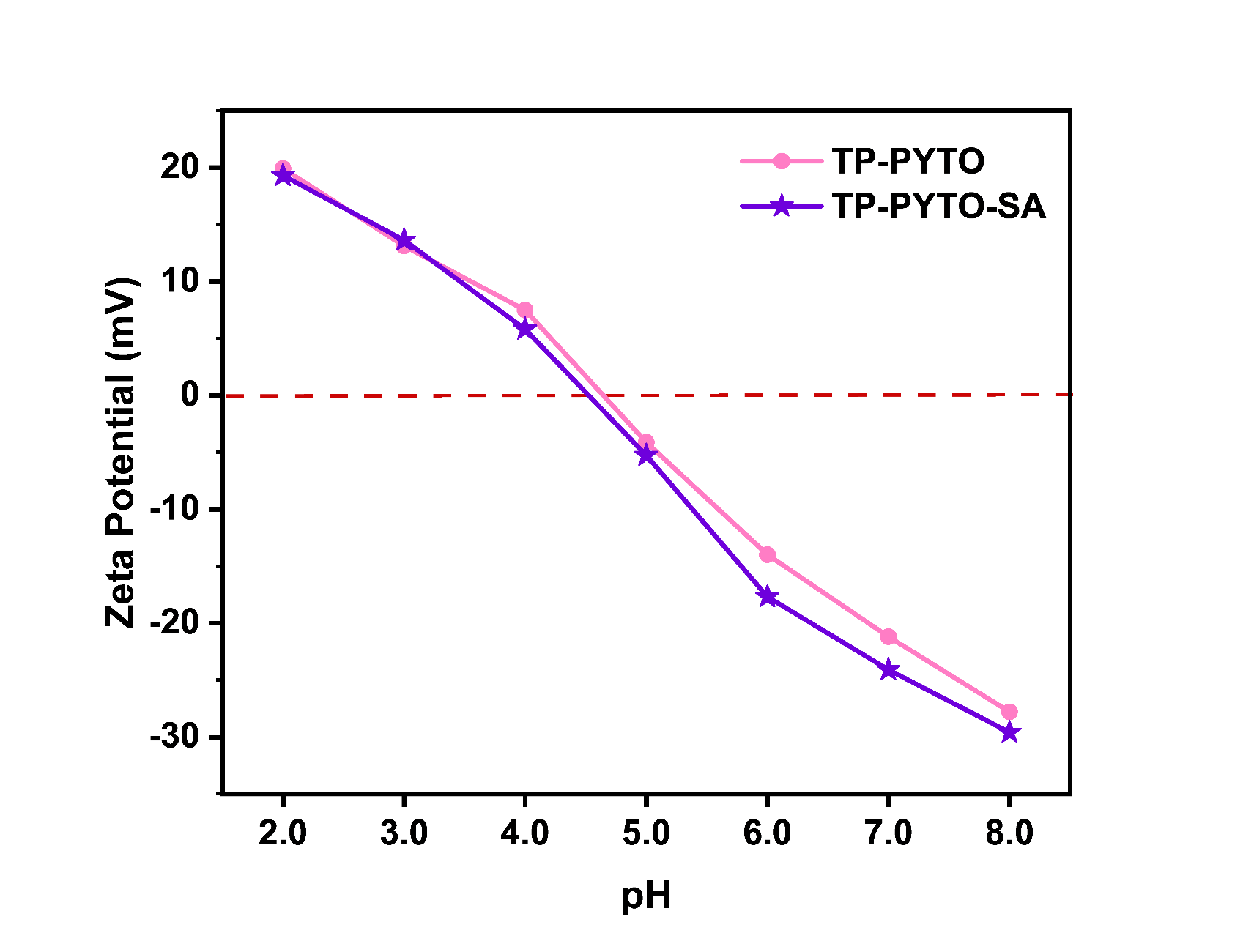


**Figure S13.** Zeta potential values of TP-PYTO and TP-PYTO-SA_20_ with different PH.

**
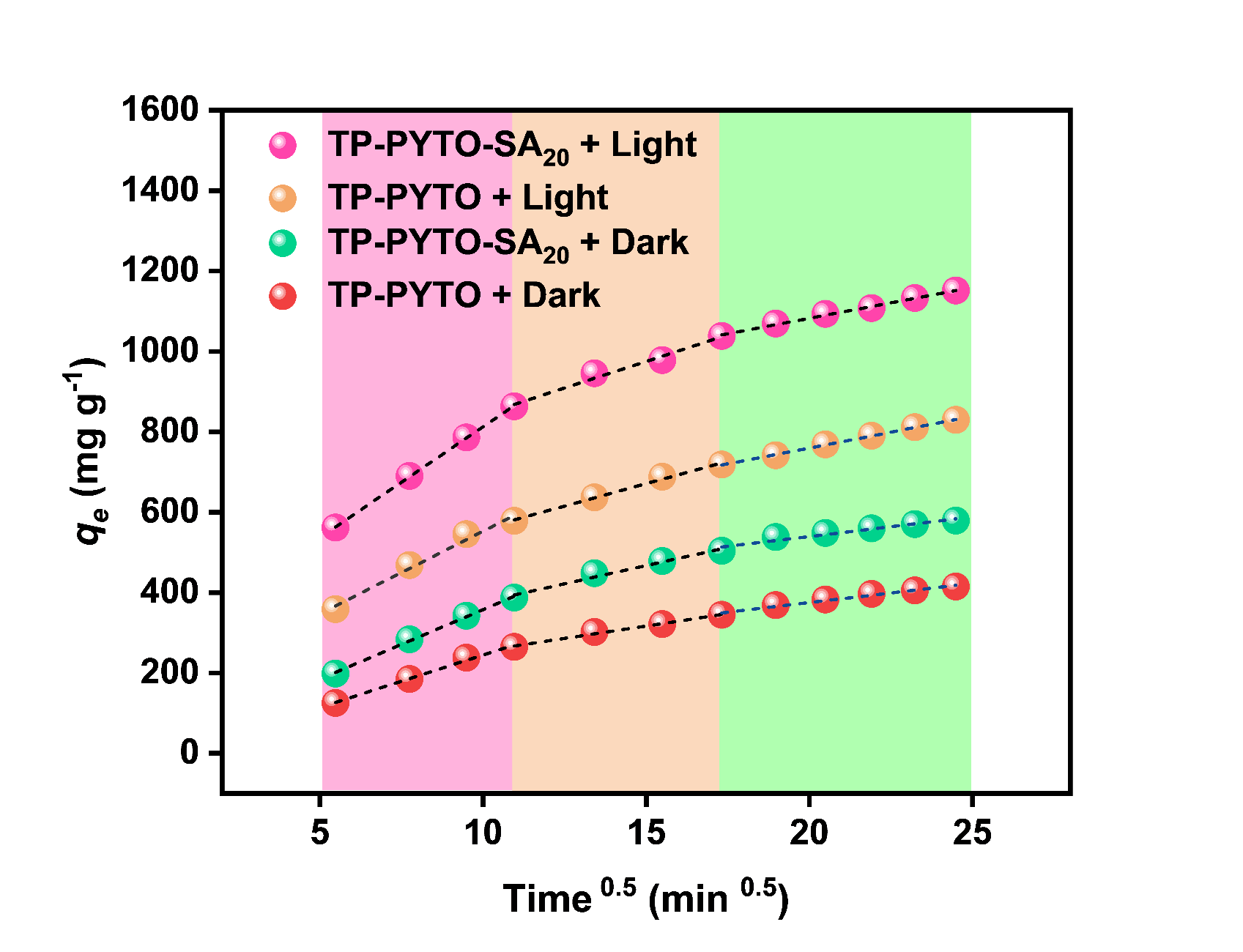
**

**Figure S14.** Intraparticle diffusion models of TP-PYTO and TP-PYTO-SA_20_ under light and dark conditions.


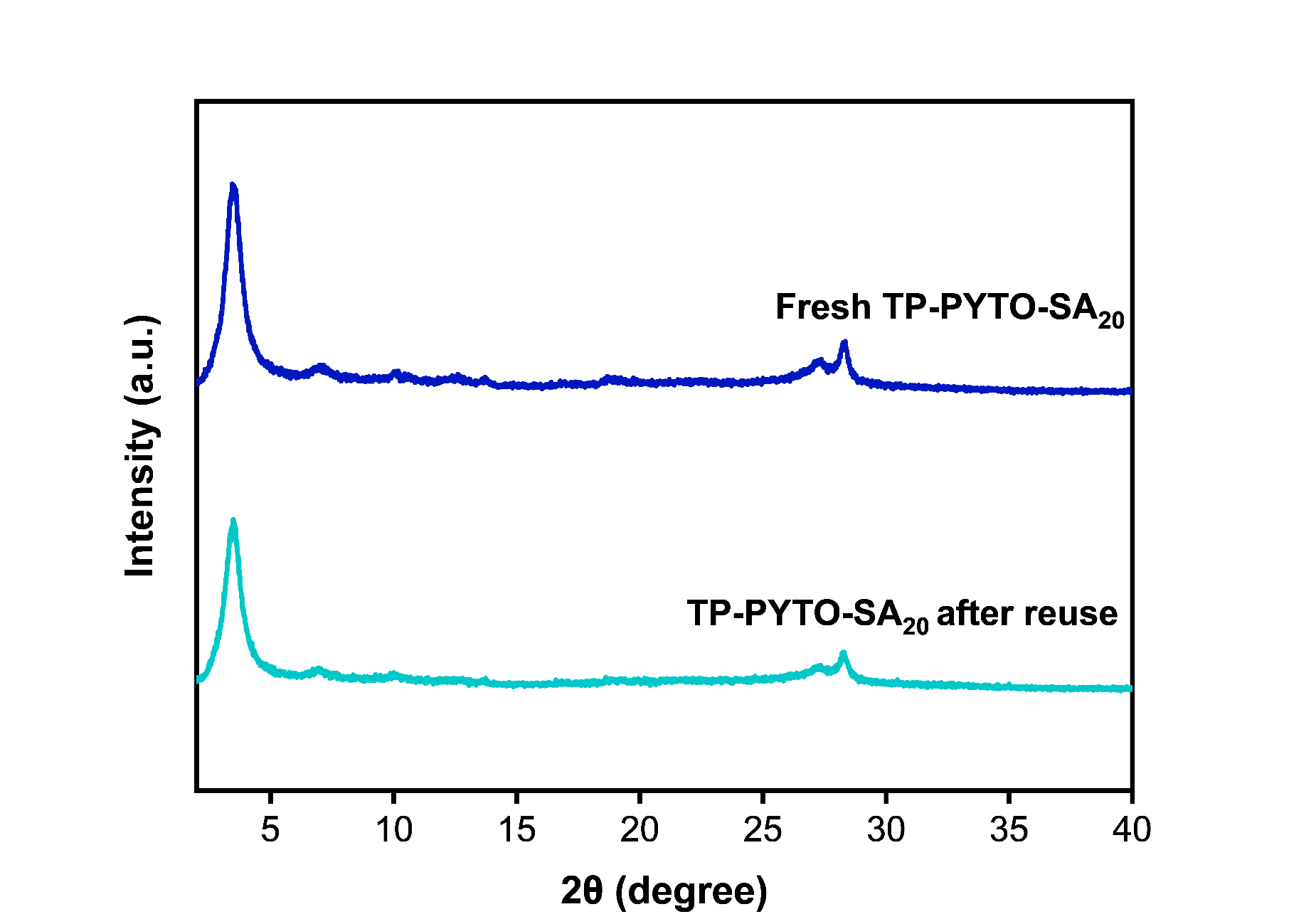


**Figure S15.** PXRD patterns of TP-PYTO-SA_20_ before and after reuse.


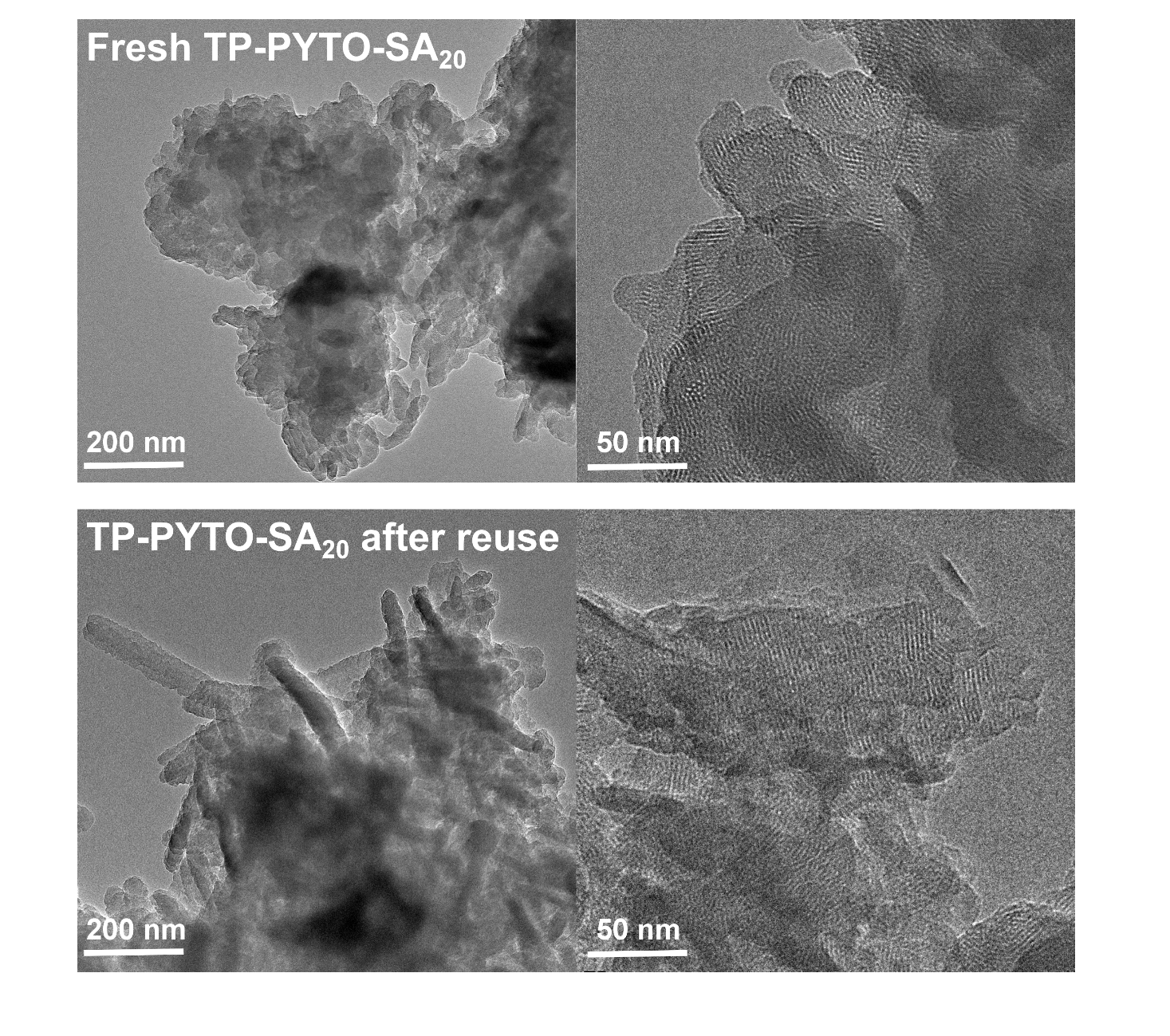


**Figure S16.** TEM image of TP-PYTO-SA_20_ before and after reuse.


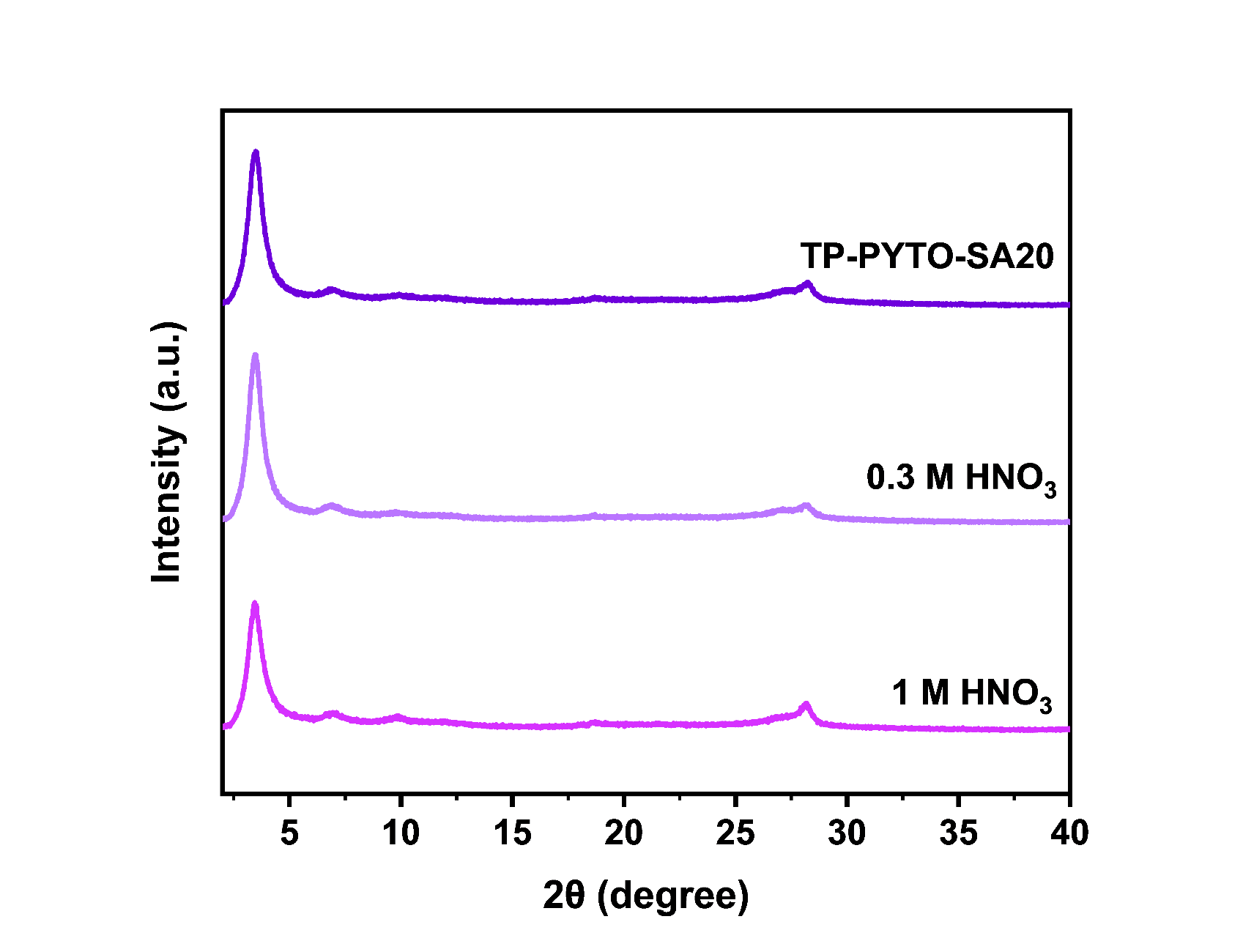


**Figure S17.** PXRD spectra of TP-PYTO-SA_20_ after treatment different concentrations of HNO_3_.


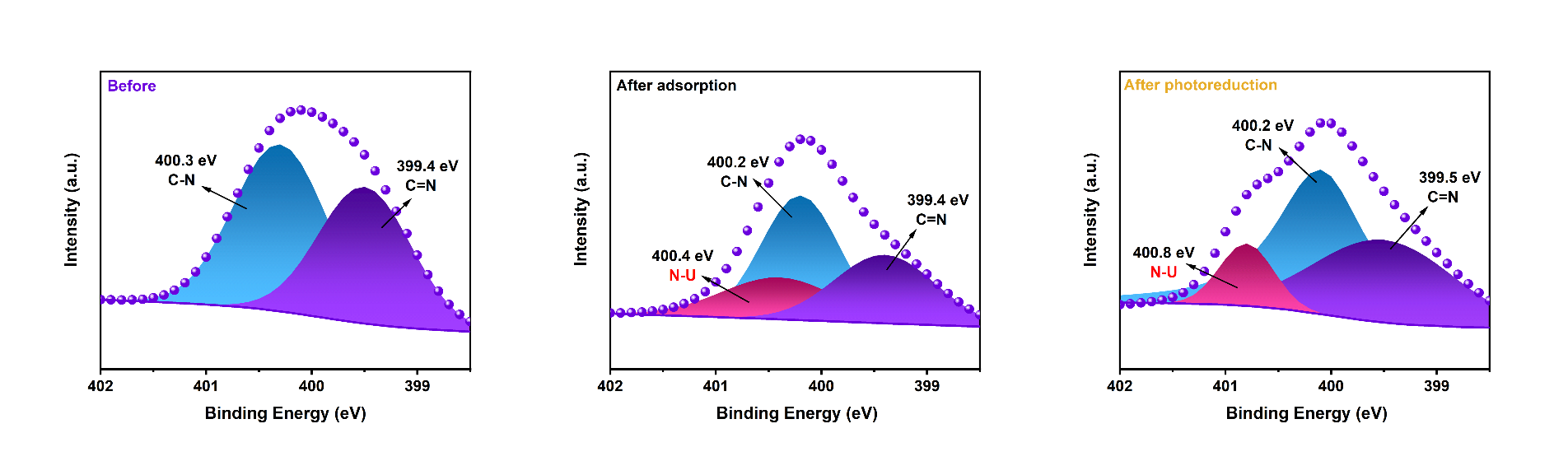


**Figure S18.** N 1s XPS spectra of TP-PYTO-SA_20_ before and after the reaction.


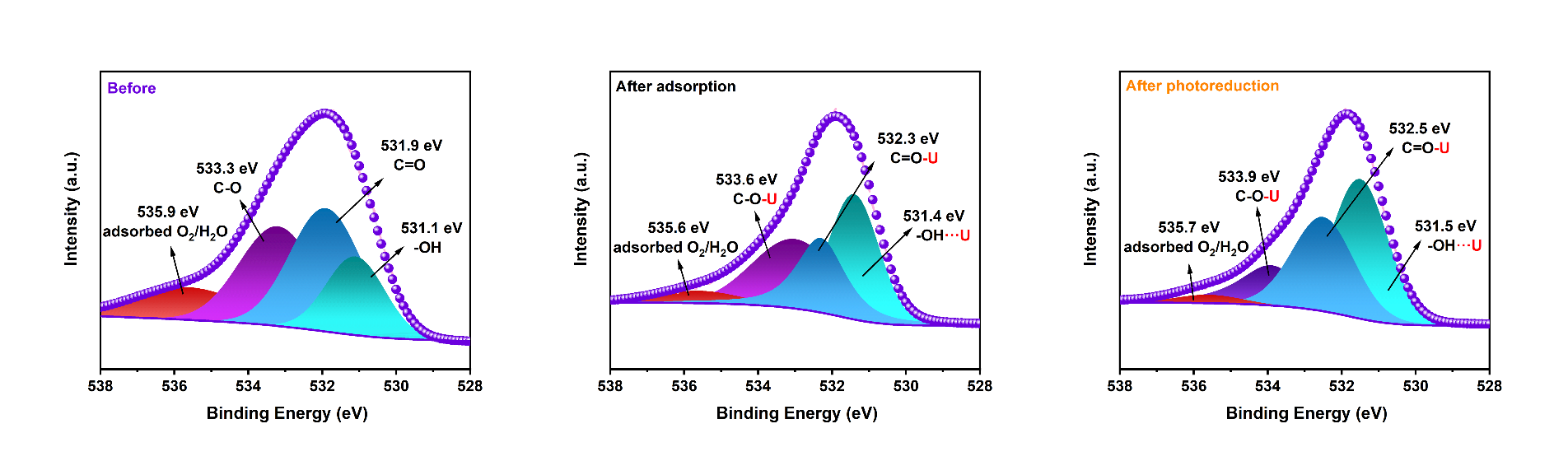


**Figure S19.** O 1s XPS spectra of TP-PYTO-SA_20_ before and after the reaction.


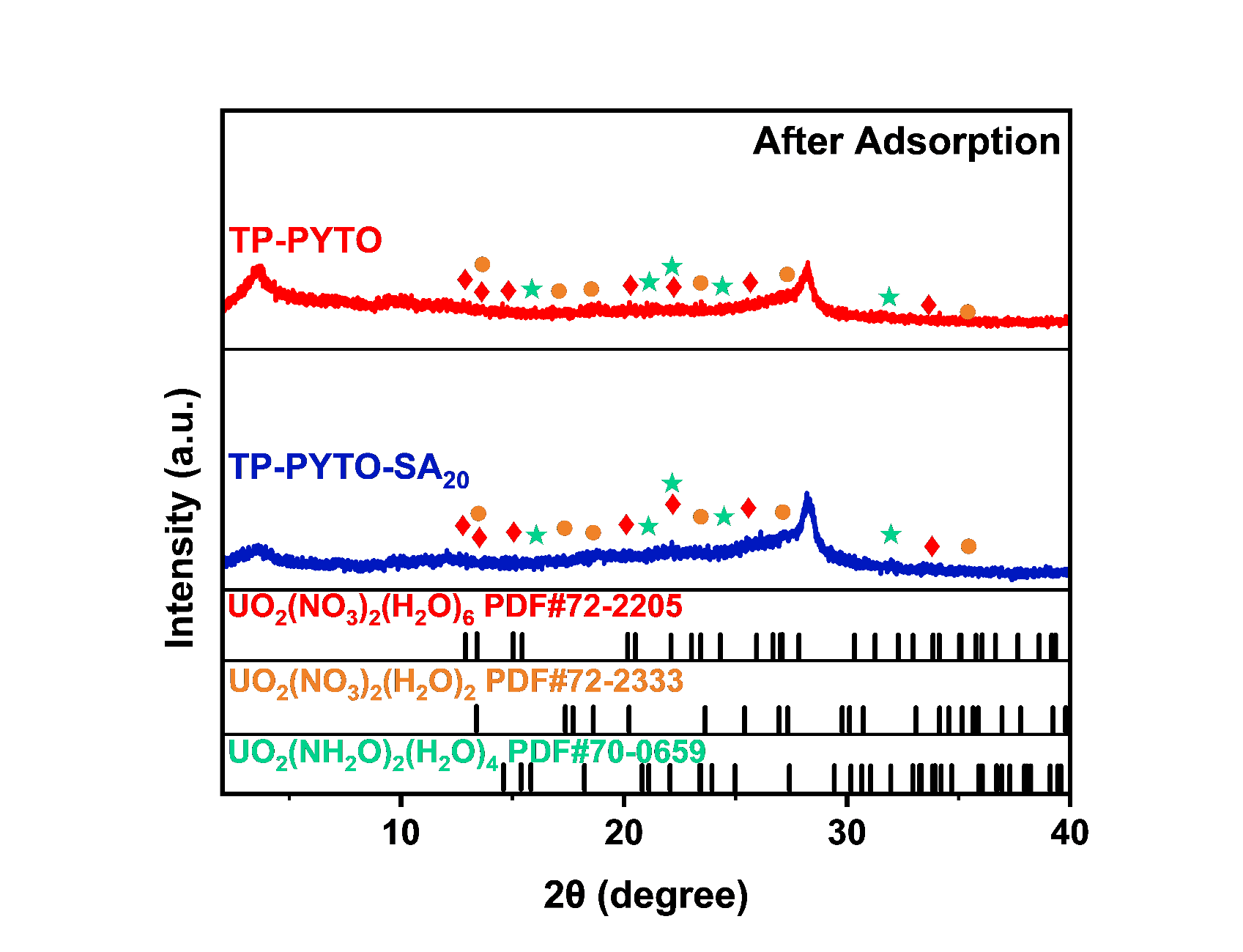


**Figure S20.** The PXRD patterns for after adsorption of TP-PYTO and TP-PYTO-SA_20_.

**
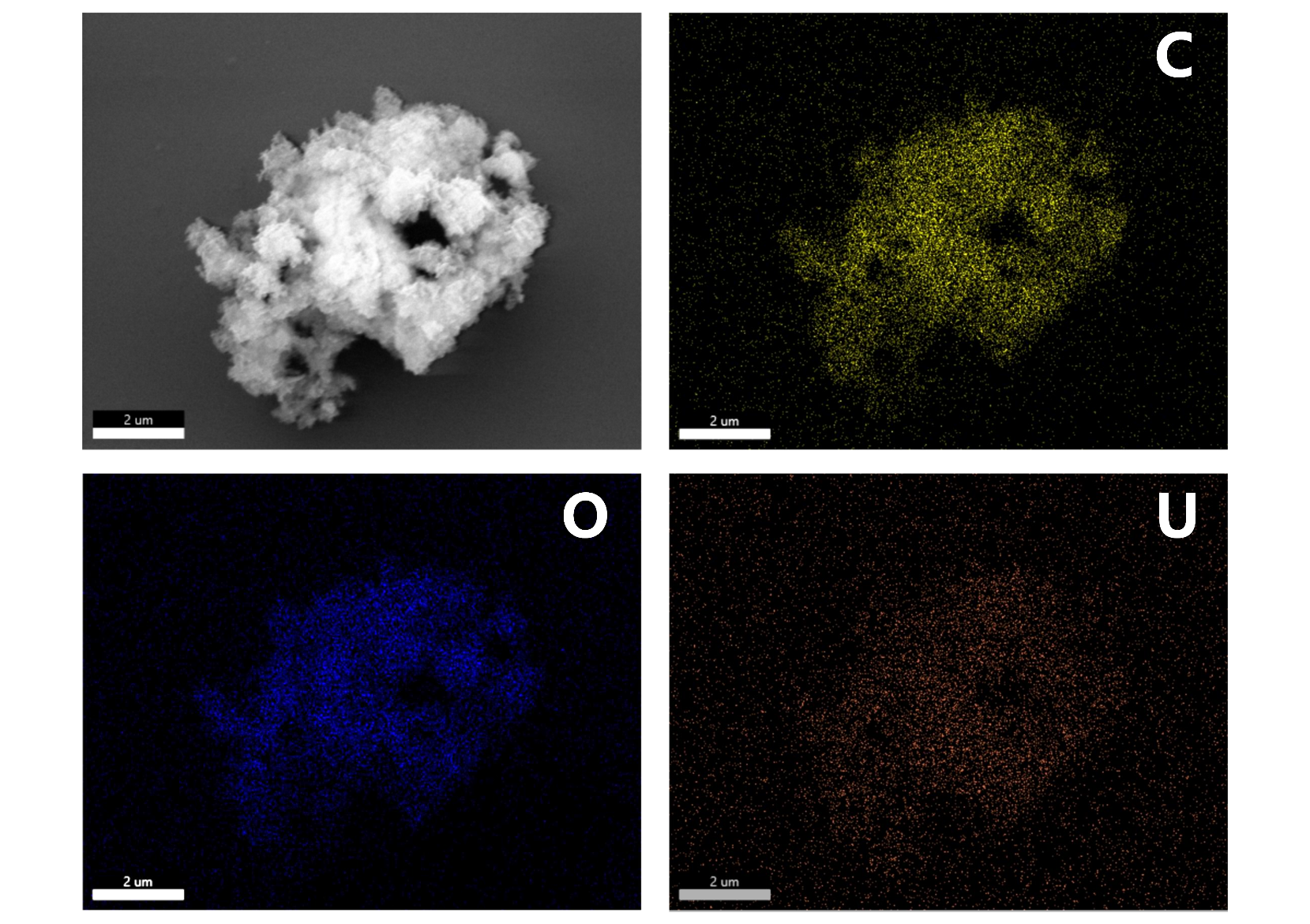
**

**Figure S21.** SEM images and EDS elemental mapping images for TP-PYTO-SA_20_ after photoreduction.


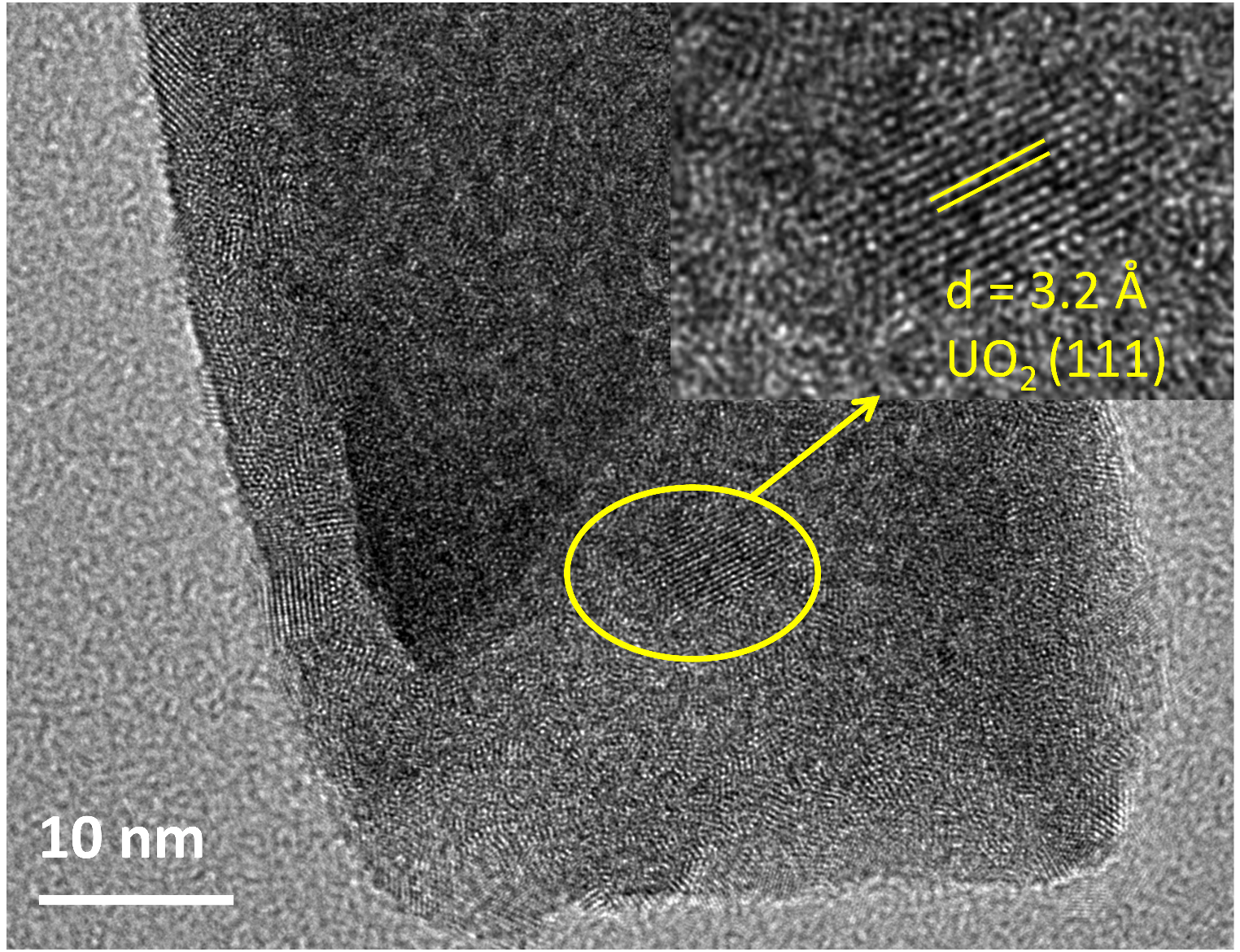


**Figure S22.** HR-TEM image of TP-PYTO-SA_20_ after photoreduction.


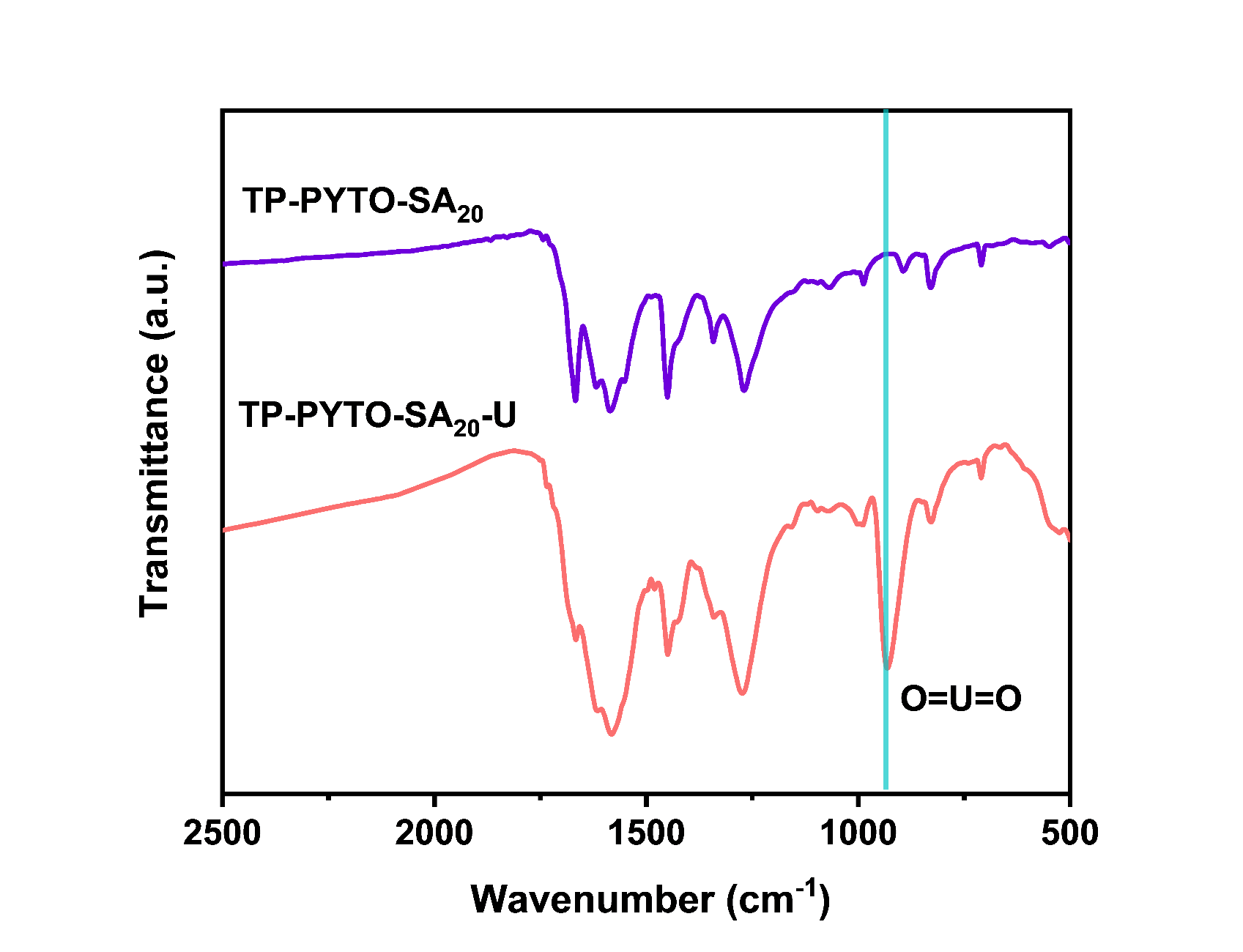


**Figure S23.** FT-IR spectra of TP-PYTO-SA_20_ after photoreaction.

**
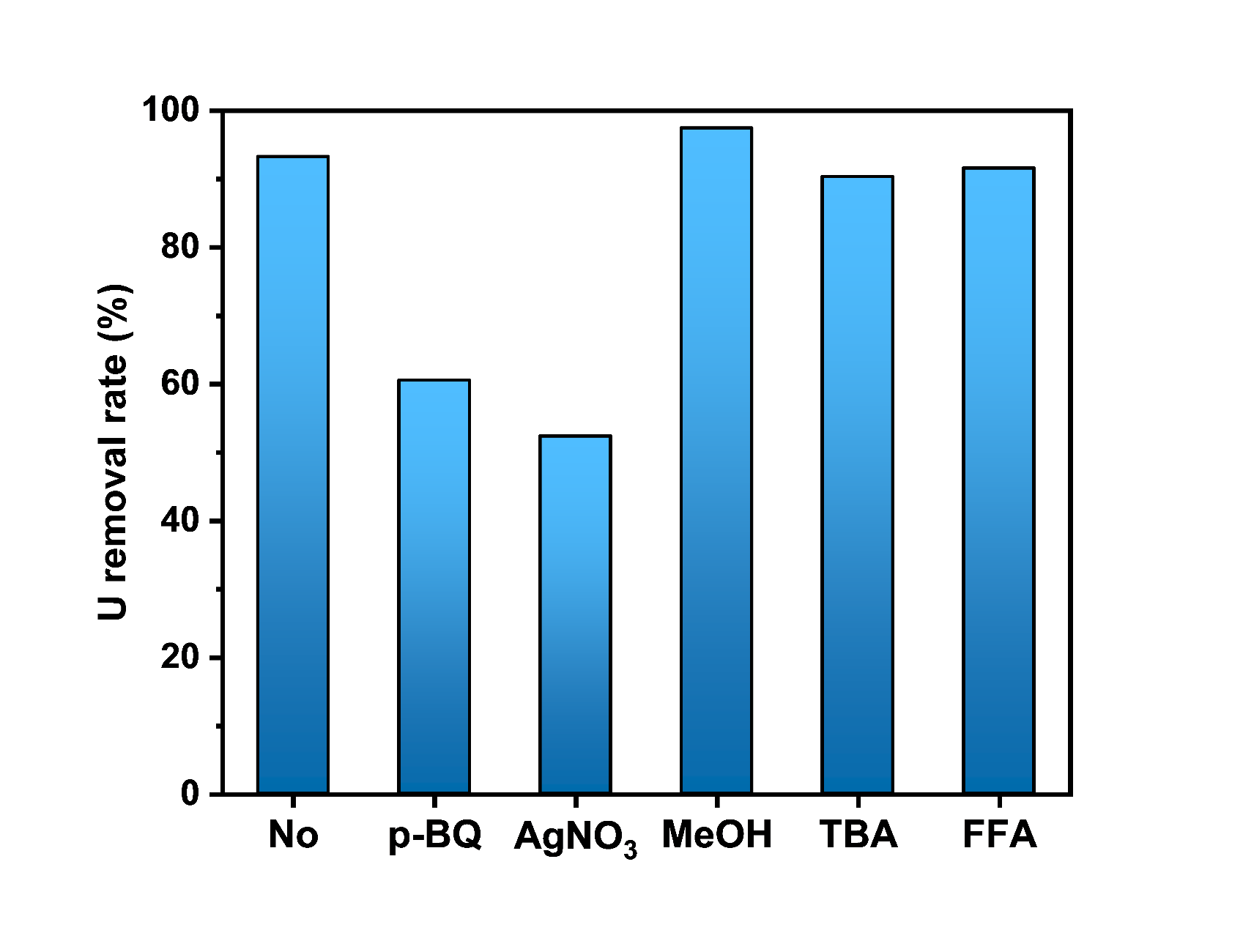
**

**Figure S24.** Effect of different radical scavengers on photoreduction of U(VI) by TP-PYTO-SA_20_.


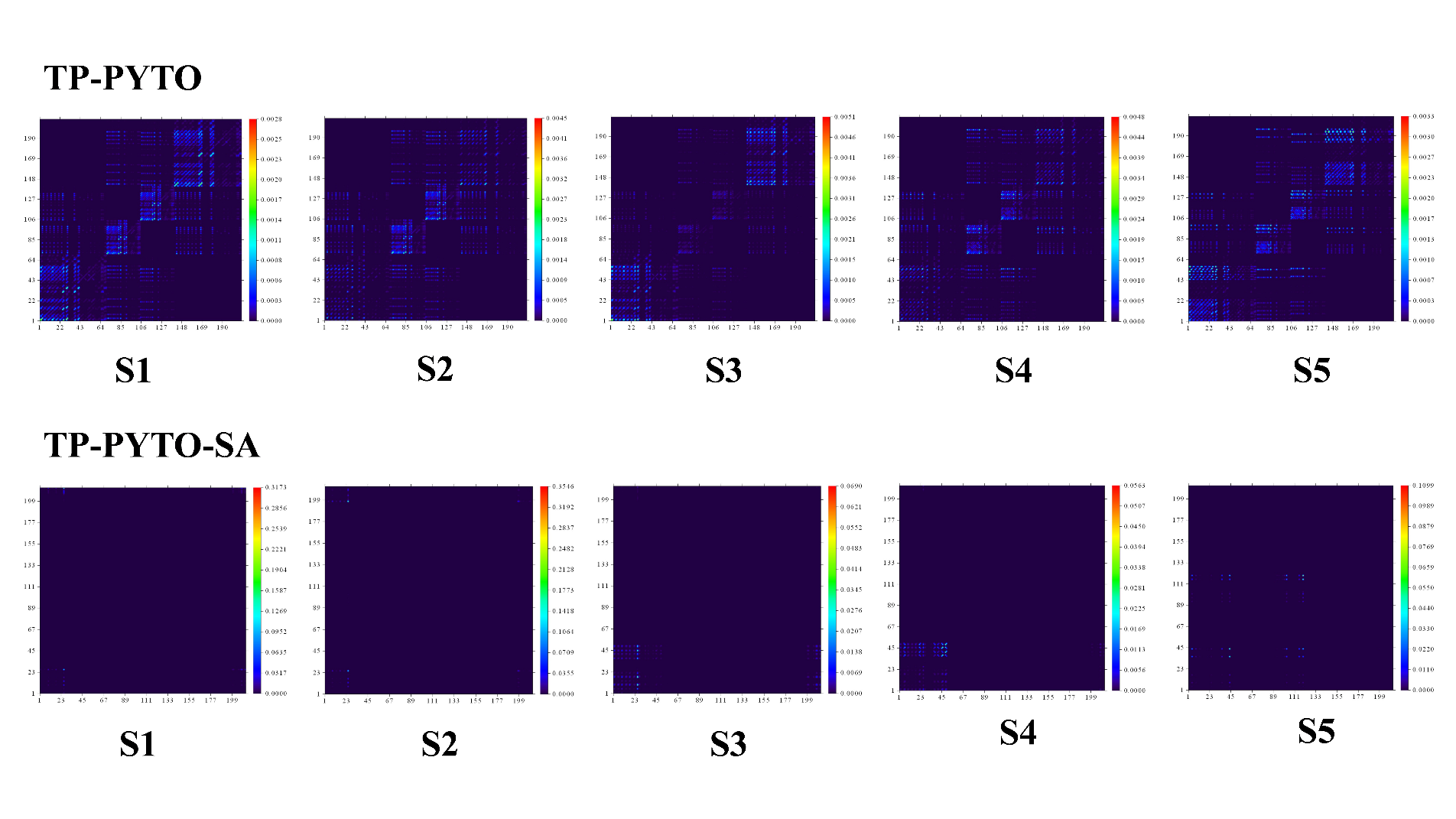


**Figure S25.** Calculated TDMs for the first five excited states of TP-PYTO and TP-PYTO-SA.

**
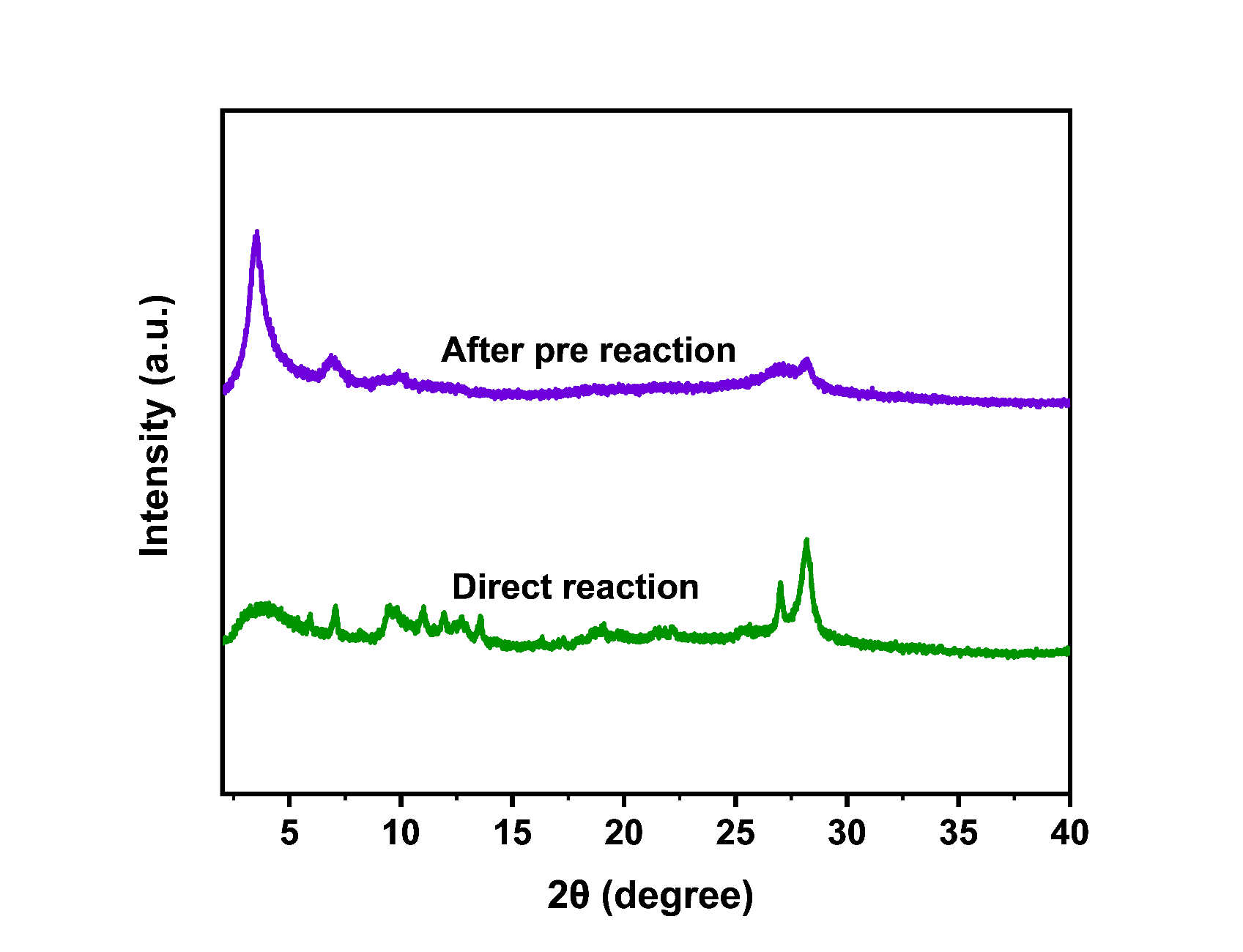
**

**Figure S26.** PXRD spectra of TP-PYTO-SA_20_ after pre reaction and direct reaction.

**Table S1.** Fractional atomic coordinates for the unit cell of TP-PYTO.

| **Space group：*P6*/m**  *a* =30.0755Å, *b*=30.0755Å, and *c*=3.5242 Å,  a=β=90°，and γ=120° | | | |
| --- | --- | --- | --- |
| **Atom list** | **x** | **y** | **z** |
| C1 | 0.97136 | 0.48363 | 0.5 |
| C2 | 0.94011 | 0.5064 | 0.5 |
| C3 | 0.88634 | 0.47527 | 0.5 |
| C4 | 0.86301 | 0.42159 | 0.5 |
| C5 | 0.89407 | 0.39924 | 0.5 |
| C6 | 0.94787 | 0.42956 | 0.5 |
| N7 | 0.80815 | 0.3886 | 0.5 |
| C8 | 0.77181 | 0.40409 | 0.5 |
| C9 | 0.7225 | 0.37007 | 0.5 |
| C10 | 0.96384 | 0.56296 | 0.5 |
| C11 | 0.97973 | 0.40479 | 0.5 |
| O12 | 0.93667 | 0.58243 | 0.5 |
| O13 | 0.95918 | 0.35806 | 0.5 |
| C14 | 0.6856 | 0.38837 | 0.5 |
| O15 | 0.70108 | 0.43234 | 0.5 |
| H16 | 0.86325 | 0.49349 | 0.5 |
| H17 | 0.87599 | 0.35769 | 0.5 |
| H18 | 0.79394 | 0.35048 | 0.5 |
| H19 | 0.78291 | 0.44437 | 0.5 |

**Table S2.** The uranium removal rate of COFs under dark and light conditions.

| COF | Removal rate (%) |
| --- | --- |
| TP-PYTO + Dark | 51.2  59.3  70.3  37.7  87.9  93.1  97.7  76.6 |
| TP-PYTO-SA_10_ + Dark |  |
| TP-PYTO-SA_20_ + Dark |  |
| TP-PYTO-SA_30_ + Dark |  |
| TP-PYTO + Light |  |
| TP-PYTO-SA_10_ + Light |  |
| TP-PYTO-SA_20_ + Light |  |
| TP-PYTO-SA_30_ + Light |  |

**Table S3.** Parameters of the Pseudo-first-order kinetics for the adsorption of uranium on COFs.

| COF | Pseudo-first-order kinetics | | | |
| --- | --- | --- | --- | --- |
|  | *q_e,expt_*  (mg g^-1^) | *q_e,calc_*  (mg g^-1^) | *R*^2^ | *K_1_* |
| TP-PYTO + Dark | 414.85 | 390.86 | 0.97593  0.98092  0.95259  0.95631 | 0.00947  0.01064  0.01422  0.01646 |
| TP-PYTO-SA_20_ + Dark | 578.76 | 552.57 |  |  |
| TP-PYTO + Light | 829.57 | 762.74 |  |  |
| TP-PYTO-SA_20_ + Light | 1151.65 | 1073.76 |  |  |

**Table S4.** Parameters of the Pseudo-second-order kinetics for the adsorption of uranium on COFs.

| COF | Pseudo-second-order kinetics | | | |
| --- | --- | --- | --- | --- |
|  | *q_e,expt_*  (mg g^-1^) | *q_e,calc_*  (mg g^-1^) | *R*^2^ | *K_2_* |
| TP-PYTO + Dark | 414.85 | 466.34 | 0.99568  0.99801  0.99007  0.99222 | 2.32×10^-5^  1.98×10^-5^  2.21×10^-5^  1.96×10^-5^ |
| TP-PYTO-SA_20_ + Dark | 578.76 | 647.64 |  |  |
| TP-PYTO + Light | 829.57 | 863.60 |  |  |
| TP-PYTO-SA_20_ + Light | 1151.65 | 1196.28 |  |  |

**Table S5.** Parameters of the Intraparticle diffusion kinetics for the adsorption of uranium on COFs.

| COF | Intraparticle diffusion kinetics | | | | |
| --- | --- | --- | --- | --- | --- |
|  | *q_e,expt_*  (mg g^-1^) | *K_ip1_* | *R*^2^ | *K_ip2_* | *R*^2^ |
| TP-PYTO + Dark | 414.85 | 26.05 | 0.99399 | 12.29  18.09  22.31  26.42 | 0.99223  0.97706  0.99459  0.98247 |
| TP-PYTO-SA_20_ + Dark | 578.76 | 34.59 | 0.99807 |  |  |
| TP-PYTO + Light | 829.57 | 41.11 | 0.98277 |  |  |
| TP-PYTO-SA_20_ + Light | 1151.65 | 55.15 | 0.99979 |  |  |

**Table S6.** Parameters of the Langmuir isotherms for the removal of uranium on COFs.

| COF | Langmuir Model | | | |
| --- | --- | --- | --- | --- |
|  | *Q_exp_*  (mg g^-1^) | *Q_max,fitted_*  (mg g^-1^) | *b*  (L mg^-1^) | *R*^2^ |
| TP-PYTO + Dark | 449.21 | 560.56 | 0.0291 | 0.99212 |
| TP-PYTO-SA_20_ + Dark | 621.64 | 734.83 | 0.0125 | 0.99206 |
| TP-PYTO + Light | 863.56 | 940.50 | 0.005 | 0.99679 |
| TP-PYTO-SA_20_ + Light | 1194.49 | 1266.57 | 0.003 | 0.99767 |

**Table S7.** Metal ions concentration of the simulated uranium-containing wastewater with pH of 5.0.

| **Metal ions** | **Initial concentration (mg L^-1^)** | **Metal ions** | **Initial concentration (mg L^-1^)** | |
| --- | --- | --- | --- | --- |
| **U** | 18.9 | **Co^2+^** | | 3.08 |
| **Mg^2+^** | 1.53 | **Ni^2+^** | | 3.30 |
| **Zn^2+^** | 3.34 | **Cu^2+^** | | 4.18 |
| **Sr^2+^** | 4.22 | **Fe^3+^** | | 5.63 |
| **Ba^2+^** | 6.18 | **Cd^2+^** | | 6.21 |

**References**

[1] M. Zhou, J. Zhou, L. Yan, S. Li, X. Cao, Y. Zhang, J. Ma, L. Shao, Z. Xie, X. Cheng, *Small.* 2025, 21, 2505330.

[2] R. Liu, D. Zhao, S. Ji, H. Shao, Y. Chen, M. Feng, T. Wang, J. Li, M. Lin, T. C. Sum, N. Yan, S. Seki, D. Jiang, *Nat. Mater.* 2025, 24, 1245.

[3] Y. Xu, F. Qiu, Y. Fu, S. F. Li, X. Su, K. Hong, M. M. Zhang, X. Zhao, Y. Wang, S. Q. Xu, *Angew. Chem. Int. Ed.* 2025, e202512603.

[4] J. Yang, Z. Zhao, X. Liu, H. Yang, J. Yang, M. Zhou, S. Li, X. Liu, X. Xu, C. Cheng, *Nat. Commun.* 2025, 16, 7654.

[5] J. Wang, M. Chen, Z. Lu, Z. Chen, L. Si, *Adv. Sci.* 2022, 9, 2203058.

[6] G. Zhou, F. Gao, T. Liu, S. Shi, H. Wang, Y. Yuan, N. Wang, *Adv. Funct. Mater.* 2024, 34, 2406329.

[7] J. Zhang, Y. Wang, L. Feng, J. Zhang, X. Tian, S. Huang, Y. Yuan, N. Wang, *Sep. Purif. Technol.* 2025, 360, 131026.

[8] S. Zhuang, R. Chen, Y. Liu, J. Wang, *J. Hazard. Mater.* 2020, 385, 121596.

[9] M. Soroushmanesh, M. Dinari, H. Farrokhpour, *Langmuir.* 2024, 40, 19073.

[10] C. Zhao, L. Sun, Y. Ai, W. Liu, *J. Clean. Prod.* 2022, 338, 130566.
